# SOX9 drives a stem-like transcriptional state and platinum resistance in high-grade serous ovarian cancer

**DOI:** 10.1172/JCI186467

**Published:** 2025-10-01

**Authors:** Alexander J. Duval, Fidan Seker-Polat, Magdalena Rogozinska, Meric Kinali, Ann E. Walts, Ozlem Neyisci, Yaqi Zhang, Zhonglin Li, Edward J. Tanner, Allison E. Grubbs, Sandra Orsulic, Daniela Matei, Mazhar Adli

**Affiliations:** 1 Department of Obstetrics and Gynecology and; 2 Driskill Graduate Program of Life Sciences, Feinberg School of Medicine at Northwestern University, Chicago, Illinois, USA.; 3 Department of Pathology & Laboratory Medicine, Cedars-Sinai Medical Center, Los Angeles, California, USA.; 4 Department of Obstetrics and Gynecology, David Gefen School of Medicine, UCLA, Los Angeles, California, USA.; 5 Department of Veterans Affairs, Greater Los Angeles Healthcare System, Los Angeles, California, USA.; 6 Robert H. Lurie Comprehensive Cancer Center, Chicago, Illinois, USA.; 7 Jesse Brown Veterans Affairs Medical Center, Chicago, Illinois, USA.

**Keywords:** Oncology, Stem cells, Cancer, Epigenetics, Transcription

## Abstract

Chemotherapy resistance remains a formidable challenge to the treatment of high-grade serous ovarian cancer (HGSOC). The drug-tolerant cells may originate from a small population of inherently resistant cancer stem cells (CSCs) in primary tumors. In contrast, sufficient evidence suggests that drug tolerance can also be transiently acquired by nonstem cancer cells. Regardless of the route, key regulators of this plastic process are poorly understood. Here, we utilized multiomics, tumor microarrays, and epigenetic modulation to demonstrate that SOX9 is a key chemo-induced driver of chemoresistance in HGSOC. Epigenetic upregulation of SOX9 was sufficient to induce chemoresistance in multiple HGSOC lines. Moreover, this upregulation induced the formation of a stem-like subpopulation and significant chemoresistance in vivo. Mechanistically, SOX9 increased transcriptional divergence, reprogramming the transcriptional state of naive cells into a stem-like state. Supporting this, we identified a rare cluster of SOX9-expressing cells in primary tumors that were highly enriched for CSCs and chemoresistance-associated stress gene modules. Notably, single-cell analysis showed that chemo treatment results in rapid population-level induction of SOX9 that enriches for a stem-like transcriptional state. Altogether, these findings implicate SOX9 as a critical regulator of early steps of transcriptional reprogramming that lead to chemoresistance through a CSC-like state in HGSOC.

## Abstract 2

The transcription factor SOX9 is sufficient to induce a stem-like transcriptional state and significant resistance to platinum treatment in high-grade serous ovarian cancer.

## Introduction

Ovarian cancer (OC) remains the deadliest gynecological cancer. The American Cancer Society estimates that approximately 20,890 women will receive an OC diagnosis, and 12,730 will succumb to the disease in 2025 (1). Chemoresistance poses a formidable challenge in the treatment of OC, particularly in its most common form, high-grade serous ovarian cancer (HGSOC) (2). Cancer cells exploit complex pathways to overcome sensitivity to first-line chemotherapies. Activation of survival/anti-apoptotic pathways, drug efflux, DNA damage repair, cell cycle checkpoints, and metabolic reprogramming are all among the hallmarks of chemoresistance (3). This state is driven by global genomic and epigenomic reprogramming (4, 5). Critically, recurrent genetic abnormalities have been associated with chemoresistance in only approximately 30% of OC cases (6, 7). This indicates that nongenetic mechanisms, such as epigenetic and transcriptional reprogramming, drive chemoresistance in a majority of cases. The drivers of this transformation, however, are poorly understood.

Understanding the molecular steps utilized by OC cells in the early stages of chemoresistance might shed critical light on the process of its acquisition and maintenance. A well-established concept of chemoresistance is the cancer stem cell (CSC) model, which postulates that chemoresistance arises in a rare population of inherently resistant stem-like cancer cells (8, 9). These cells are transcriptionally more plastic and have a higher propensity to survive chemotherapy treatment and initiate tumors (10). Indeed, these cells are often referred to as tumor initiating cells. A complementary model is one in which most chemotherapy-treated cancer cells have the capacity to reprogram their transcriptomes to better survive treatment (11). This model postulates that tumor cells do not necessarily develop within a classical Darwinian evolution model, but rather within a Lamarckian framework. In other words, traits are induced to develop in heterogeneous populations of nonadapted lineages over nonreplicative timescales (12). Several reports have indicated transcriptional heterogeneity among chemotherapy-naive cells as an important source of chemoresistance, suggesting that tumor-wide plasticity could also play a key role (13, 14). Interestingly, lineage tracing and single-cell sequencing experiments have highlighted that the overall transcriptional heterogeneity and malleability are induced by drug treatment, persist in resistant cells, and allow these cells to quickly adapt and respond to their new environment (15–17).

To study nongenetic drivers of chemoresistance, we identified super-enhancers and their target genes that are commissioned specifically in resistant cells (18). Super-enhancers, i.e., clusters of closely associated enhancers, are known to regulate critical transcription factors (TFs) and developmental regulators that dictate cellular and lineage identity in normal development as well as in cancer (19, 20). Notably, master regulator TFs, which are often associated with super-enhancers, are recognized as critical drivers of tumorigenesis in HGSOC (21–24). In this study, we investigate how the TF sry-box 9 (SOX9), which we have identified as a resistant state–specific, super-enhancer–regulated TF (18), drives chemoresistance in HGSOC. SOX9 is a high-mobility group box TF that is vital to developmental pathways including sex determination, chondrogenesis, and hair follicle stem-niche maintenance (25, 26). Recently, increasing evidence has implicated SOX9 in cancer pathogenesis. Specifically, SOX9 has been found to influence self-renewal, invasion, and proliferation in lung (27), hepatocellular (28), breast (29), prostate (30), skin (31), and pancreatic cancers (32). In a recent study, SOX9 was characterized as a pioneer factor that can perform fate-switching in hair follicle stem cells through global changes in chromatin structure (33). In another, SOX9 and SOX2 expression were found to be associated with each other as well as with poor clinical outcomes in HGSOC clinical samples (34).

In this study, we provide evidence supporting the hypothesis that SOX9 drives chemoresistance in HGSOC by reprogramming the transcriptional program of naive OC cells into stem-like cancer cells. We performed bulk- and single-cell sequencing, immune staining of primary tissues, and epigenetic modulation of endogenous loci and show that SOX9 is not only necessary for chemoresistance in OC, but that its expression is sufficient for its acquisition. Mechanistically, our genetic and epigenetic perturbations and single-cell multiomic profiling of naive tumors show that SOX9 expression reprograms the global transcriptional program into a stem-like transcriptional state and cellular phenotype, indicating that SOX9 is a regulator of OC stem cells and is a driver of chemoresistance in HGSOC.

## Results

### SOX9 is amplified in OC, and its expression is further induced by platinum treatment.

**Figure 1:**
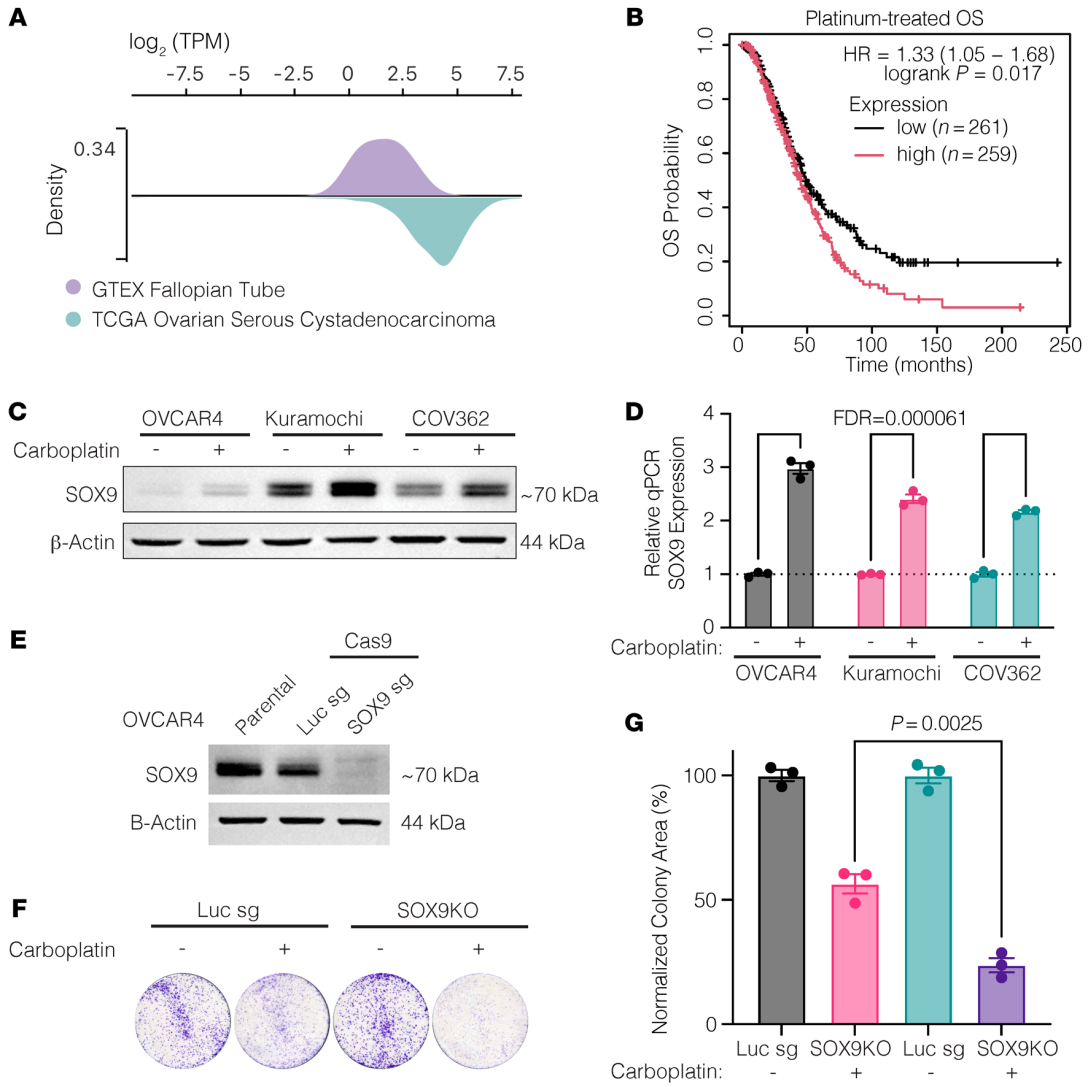
*SOX9* is overexpressed, is a poor prognostic indicator, and is induced by acute platinum exposure in OC. (**A**) *SOX9* expression is shown in transcripts per million (TPM) between normal fallopian tube tissue from the Genotype-Tissue Expression database and in ovarian serous cystadenocarcinoma from The Cancer Genome Atlas. (**B**) Overall survival (OS) is shown between *SOX9* low- and high-expressing patients (Q1 vs. Q4) who received platinum treatment. HR, hazard ratio; *P* value was from a log-rank test. (**C**) Western blot showing SOX9 in OVCAR4, Kuramochi, and COV362 OC cell lines before and after receiving 72 hours of 20 μM carboplatin treatment. (**D**) Reverse transcription quantitative PCR showing relative *SOX9* expression in the same cells as in **C**. Analysis was performed in both biological and technical triplicates. Values are shown as mean ± SEM; *n* = 3. Significance was calculated using multiple 2-tailed paired Student’s *t* tests with Benjamini, Krieger, and Yekutieli correction for multiple comparisons. (**E**) Western blot depicting SOX9 knockout in OVCAR4 cells. (**F**) Example wells of colony formation assay showing colony growth of OVCAR4-Cas9 cells with either a luciferase sgRNA or a SOX9-KO sgRNA untreated or treated with 20 μM carboplatin. (**G**) Quantification of normalized percent colony coverage of colony formation assay seen in **F**. Values are shown as mean ± SEM; *n* = 3. *P* value was calculated using a 2-tailed Student’s *t* test.

Recent histological staining and whole-exome sequencing data have highlighted the epithelium of the fallopian tube as the likely cell of origin for HGSOC (35, 36). We initially assessed whether SOX9 expression is significantly higher in HGSOC tumors compared with the fallopian epithelium. Using HGSOC tumor data from The Cancer Genome Atlas and normal fallopian tube epithelium (FTE) expression data from the Genotype-Tissue Expression database (37) in the UCSC Xena platform (38), we determined that SOX9 expression is much higher in HGSOC tissues than in normal FTE (Figure 1A). Notably, the platinum-treated patients in the top quartile of SOX9 expression in an integrated microarray database (*n* = 259) had a significantly shorter overall survival probability than patients in the bottom quartile (*n* = 261) (hazard ratio = 1.33; log-rank *P* = 0.017) (Figure 1B) (39). Importantly, treatment of HGSOC cell lines (OVCAR4, Kuramochi, and COV362) with carboplatin — the most common first-line chemotherapy for HGSOC — resulted in acute and robust SOX9 induction at both RNA and protein levels within 72 hours (Figure 1, C and D), suggesting that SOX9 might be critical for the early response to platinum treatment.

### SOX9 ablation leads to increased platinum sensitivity.

To understand SOX9’s role in the HGSOC response to platinum therapy, we knocked out the gene using a SOX9-targeting sgRNA and CRISPR/Cas9 (Figure 1E). Knocking out SOX9 at a population level significantly increased the cells’ sensitivity to carboplatin treatment, as measured by a colony formation assay (2-tailed Student’s *t* test, *P* = 0.0025) (Figure 1, F and G). In the absence of chemotherapy, SOX9-depleted cells also had an accelerated growth rate compared with parental cells, as measured by an Incucyte live-cell imager (Supplemental Figure 1; supplemental material available online with this article; https://doi.org/10.1172/JCI186467DS1).

### SOX9 expression is induced after chemotherapy in patient samples.

**Figure 2:**
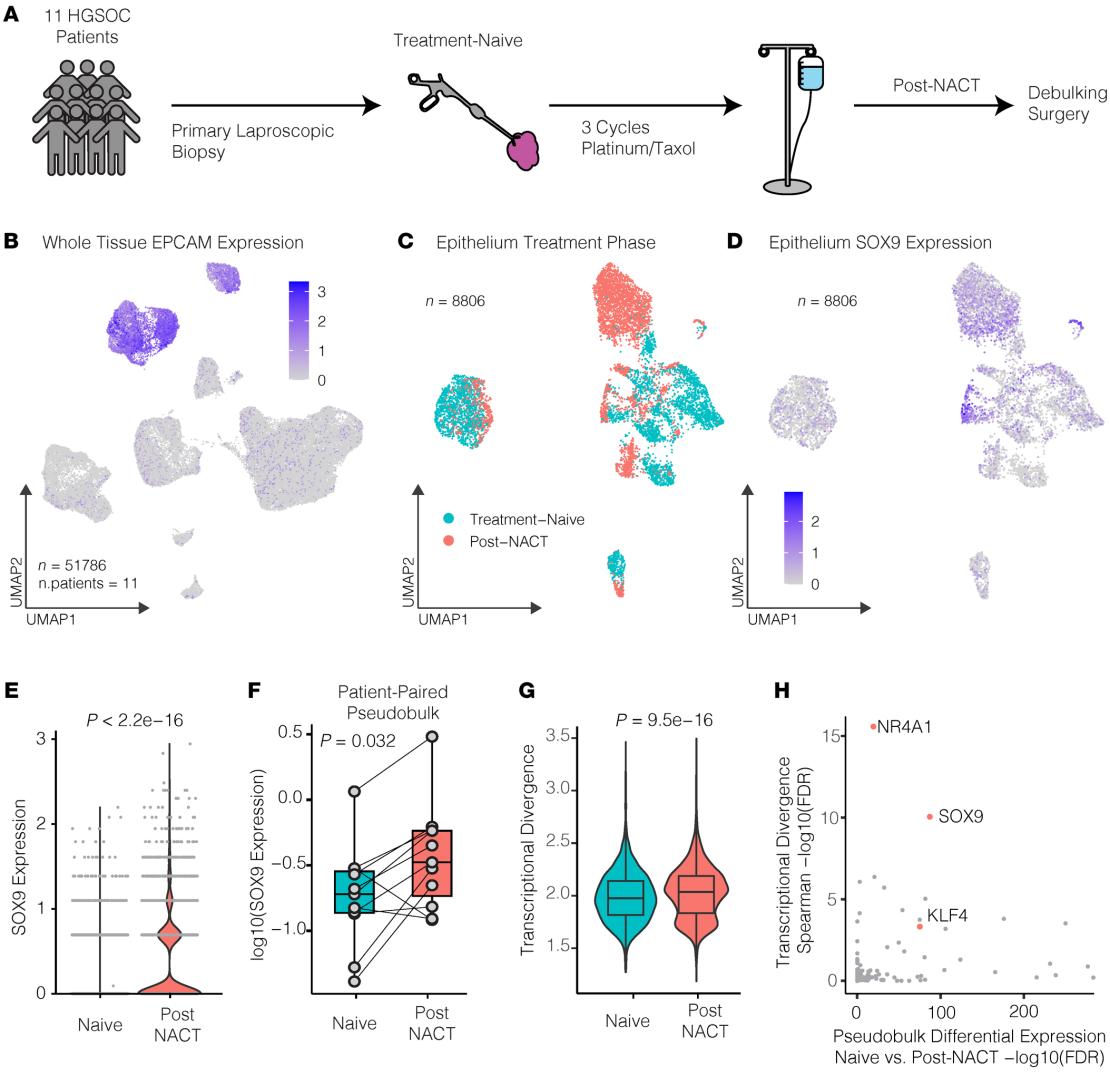
SOX9 is induced in HGSOC patients following platinum treatment and is highly associated with transcriptional malleability. (**A**) Schematic overview depicting when samples were collected from patients. (**B**) Uniform Manifold Approximation and Projection (UMAP) feature plot showing *EPCAM* expression in a longitudinal scRNA-Seq dataset representing HGSOC tumors from 11 patients consisting of 51,786 total cells. (**C**) UMAP plot showing epithelial cells only from the longitudinal dataset color coded for treatment-naive and post-NACT cells. (**D**) UMAP feature plot showing *SOX9* expression in epithelial cells in the longitudinal dataset. (**E**) Violin plot showing *SOX9* expression in epithelial cells in treatment-naive versus post-NACT cells. *P* value was calculated using Wilcoxon’s signed-rank test. (**F**) Pseudo-bulk, patient-paired box plot of *SOX9* expression in epithelial cells in treatment-naive versus post-NACT cells. *P* value was calculated using a paired Wilcoxon’s signed-rank test. (**G**) Violin plot showing transcriptional divergence per epithelial cell separated between treatment-naive versus post-NACT cells. *P* value was calculated using Wilcoxon’s signed-rank test. (**H**) Scatterplot showing –log10(FDR) for the Spearman’s rank correlation between epithelial transcriptional divergence and TF expression as well as differential expression between treatment-naive versus post-NACT cells. Box-and-whisker plots depict median, interquartile range, and data range.

We next studied whether the chemotherapy-induced SOX9 upregulation is recapitulated in primary tumor samples. To this end, we utilized a publicly available longitudinal single-cell RNA-Seq (scRNA-Seq) dataset of 11 patient HGSOC tumors that were profiled before and after 3 cycles of platinum/taxane neo-adjuvant chemotherapy (NACT) (Figure 2A) (40). Of the 51,786 cells in the dataset, 8,806 had been previously identified by Zhang et al. as epithelial cancer cells based on their expression of WFDC2, PAX8, and EPCAM (Figure 2B) (40). When grouping these cells by treatment status (treatment-naive or post-NACT), we found that SOX9 is upregulated consistently in post-NACT cells (Figure 2, C and D). To assess whether this increase in SOX9 expression is found in every cell and patient tumor, we plotted it at a single-cell (Figure 2E) and patient-specific pseudo-bulk RNA level (Figure 2F). At both levels, SOX9 expression was significantly increased in the post-NACT tissues (Wilcoxon’s *P* < 2.2e-16 and Wilcoxon’s paired *P* = 0.032, respectively). SOX9 expression increased in 8 of the 11 patients after chemotherapy, supporting the in vitro findings that SOX9 is chemotherapy induced and could be a critical transcriptional driver of chemoresistance.

### SOX9 expression is associated with transcriptional divergence, an indicator of stemness and plasticity.

A hallmark of chemoresistance, particularly nongenetic resistance, is transcriptional plasticity (41–43). We aimed to assess SOX9’s association with increased transcriptional plasticity in the patient samples across treatment phases. To this end, we utilized a measurement called transcriptional divergence, which was first described by Virk et al. as a metric for measuring overall transcriptional malleability (44). This metric is defined as the sum of the expression of the top 50% of detected genes divided by the sum of the expression of the bottom 50%, or the P50/P50, which represents a cell’s ability to respond effectively to external stressors such as chemotherapy and is amplified in stem and CSCs where overexpressed genes are amplified and lowly expressed genes are inhibited (44). Transcriptional divergence has been shown to be a poor prognostic indicator for patient survival in lung, breast, and colon cancers (44). First, we calculated the P50/P50 value for every epithelial cell in the longitudinal scRNA-Seq dataset. We found that transcriptional divergence is significantly increased in these cells following NACT (Wilcoxon’s *P* = 9.5e-16) (Figure 2G). To understand which transcriptional regulators might be affecting plasticity, we calculated the Spearman’s correlation coefficient between transcriptional divergence and the expression of every TF (*n* = 1,639 TFs). This unbiased analysis identified SOX9 as one of the highest-ranking TFs associated with overall transcriptional divergence (ρ = 0.147, Wilcoxon’s FDR = 6.3e-18) (Figure 2H). This is notable because SOX9 ranked above other well-described oncogenic stem factors such as KLF4. Even when compared with the factor that ranked above it (NR4A1), SOX9 was more significantly upregulated following NACT. These findings suggest that SOX9 is a more critical determinant of transcriptional malleability than even canonical stem factors in primary HGSOC cells.

### Chemo-treated HGSOC tumors have higher nuclear SOX9 levels.

**Figure 3:**
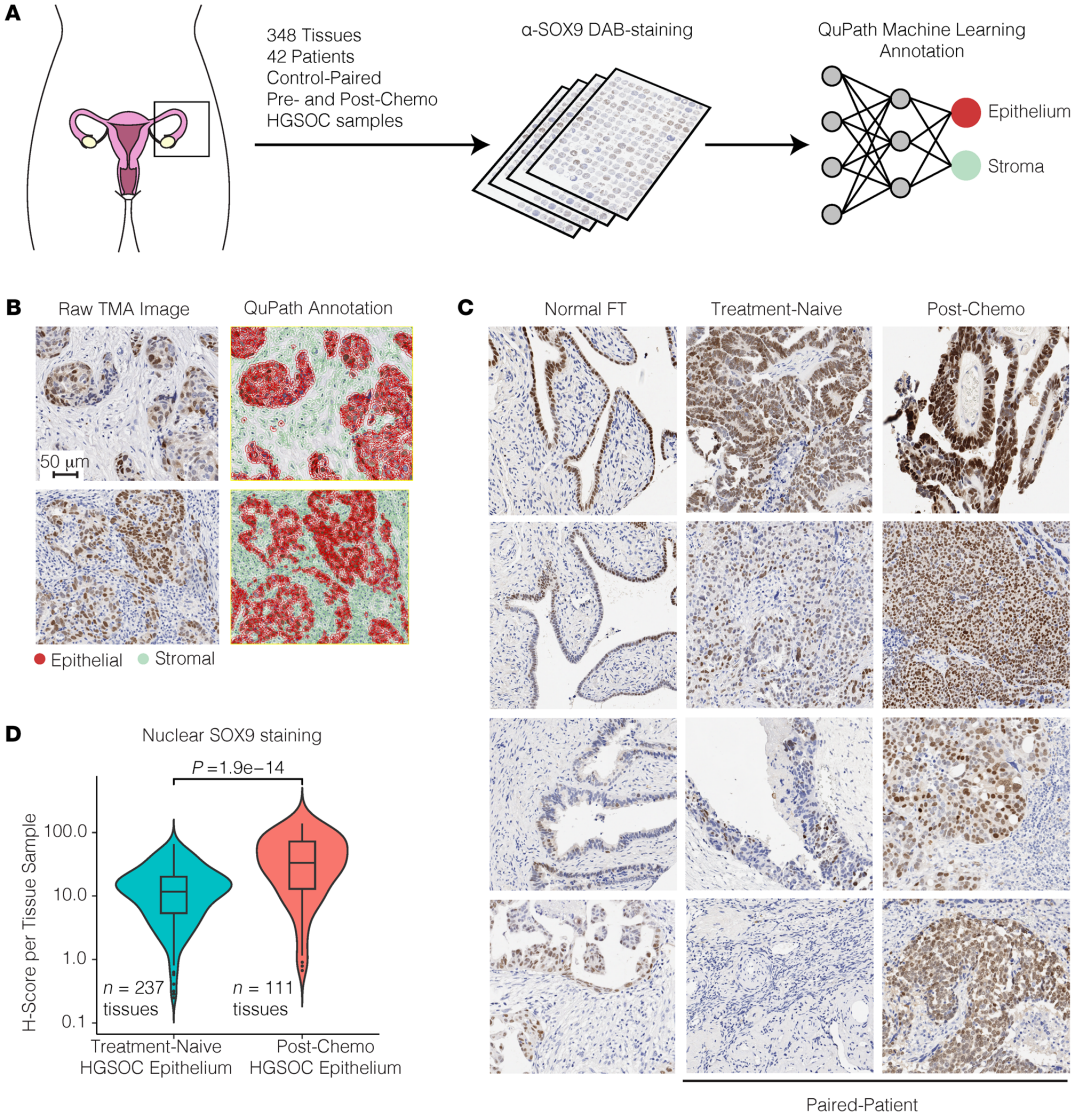
Tumor microarrays (TMAs) show nuclear SOX9 levels increase significantly in HGSOC epithelial cells following NACT. (**A**) Schematic overview depicting experimental design. (**B**) Representative images showing 2 HGSOC tissues within TMAs and their morphology-based annotations. Red, epithelial cells; green, stromal cells. Scale bar: 50 μm. (**C**) Representative images showing either normal fallopian tube (FT) tissue or patient-paired (4 patients shown) treatment-naive and post-NACT HGSOC tumor tissue. (**D**) Violin plot showing h-scores for all HGSOC epithelial cells in every intact tissue punch across both TMA slides. Data are separated by treatment status. *P* value was calculated using Wilcoxon’s ranked-sum test. Box-and-whisker plot depicts median, interquartile range, and data range.

We next aimed to verify the findings from the scRNA-Seq data at the protein level using a diverse panel of patient tumors. To this end, we utilized 2 HGSOC tissue microarrays (TMAs) containing 348 paired HGSOC tissues from 42 patients before and after chemotherapy treatment (Figure 3A) and normal FTE tissue (45). The SOX9 immunohistochemical signal intensity from the TMAs was analyzed using QuPath software. We first identified individual cells and nuclei before classifying cells as either epithelial or stromal using a machine learning model trained on cell morphology (Figure 3B) (46). This analysis revealed 2 major insights. First, we found significantly higher SOX9 protein levels within epithelial nuclei and cytoplasm compared with nonepithelial cells, as assessed by the h-score of the tissues (calculated using mean nuclear/cytoplasmic DAB staining) (Supplemental Figure 2, A and B). Second, we found that both nuclear and cytoplasmic SOX9 protein levels were significantly induced in the epithelium after chemotherapy (Figure 3, C and D, and Supplemental Figure 2C), confirming the scRNA-Seq findings that SOX9 is induced in tumors following chemotherapy.

### Multiomics analysis of an epithelial-enriched tumor enables the discovery of SOX9-enriched stem-like cells.

**Figure 4:**
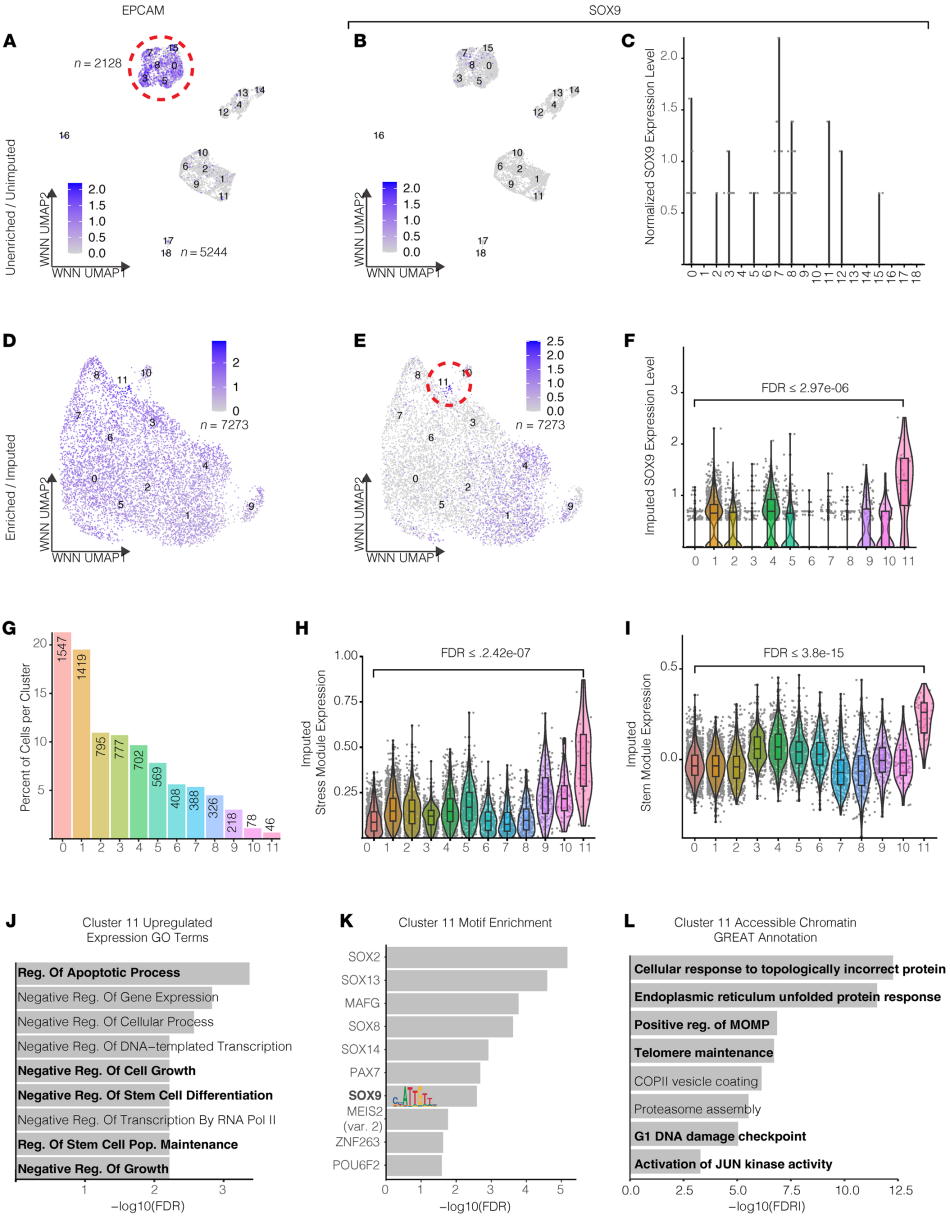
Epithelial cell enrichment and single-cell imputation identify *SOX9*-enriched CSC population in multimodal treatment-naive HGSOC patient tumor dataset. (**A**) UMAP plot showing whole-tissue WNN clustering for treatment-naive HGSOC patient tumor using both scRNA- and ATAC-seq and showing EPCAM expression. (**B**) UMAP feature plot showing *SOX9* expression within whole-tissue HGSOC multimodal dataset from **A**. (**C**) Violin plot depicting *SOX9* expression within whole-tissue HGSOC multimodal dataset from **A**. (**D**) UMAP plot showing epithelium-enriched tissue WNN clustering for treatment-naive HGSOC patient tumors using both scRNA- and ATAC-seq and showing EPCAM expression. (**E**) UMAP feature plot showing *SOX9* expression within epithelium-enriched HGSOC multimodal dataset from **D**. (**F**) Violin plot depicting *SOX9* expression within epithelium-enriched tissue HGSOC multimodal dataset from **D**. (**G**) Number of cells per cluster in the same patient sample as in **D**. (**H** and **I**) Violin plots showing relative stress module expression, and OC stem cell module expression per cluster in same patient sample as in **D**. Significance values are Wilcoxon’s signed-rank FDR. (**J**) GO terms enriched in cluster 11 transcriptional data of the patient sample in **D**. Data were plotted with –log10(FDR). (**K**) Motif enrichment in cluster 11 of the patient sample in **D**. Motif statistics are shown along with –log10(Bonferroni-adjusted *P* value). (**L**) GREAT analysis for chromatin fragments found in cluster 11 of the patient sample in **D**. Data are plotted with –log10(FDR). Box-and-whisker plots depict median, interquartile range, and data range.

Our IHC data showed the presence of high SOX9-expressing epithelial cells in untreated tumors. However, we could not determine if these cells also resemble stem-like cancer cells based on these data alone. We reasoned that if SOX9 is important for transcriptional plasticity maintenance/induction, it should be identifiable in the CSCs present in chemo-naive tumors. Therefore, we sought to characterize CSCs in a naive HGSOC tumor using multimodal scRNA-Seq and assay for transposase-accessible chromatin sequencing (ATAC-Seq). To this end, we performed 10X Genomics Multiome sequencing on a whole-tissue HGSOC tumor (Figure 4, A–C). Using weighted nearest neighbor (WNN) clustering (which utilizes both expression and accessibility datasets), we identified 18 distinct clusters, each associated with unique expression and chromatin states (Figure 4, A and B, and Supplemental Figure 3). Among these, 6 clusters were identified as epithelial (through their expression of the marker Epithelial cell adhesion molecule [EPCAM]). Notably, however, none of these clusters appeared to have significant *SOX9* expression (Figure 4, B and C). CSCs represent only approximately 0.01%–2% of the total cell mass in a tumor (47). This low cell number, combined with the high number of dropouts in single-cell sequencing, creates a formidable challenge to detecting CSCs. To overcome this challenge, we reperformed single-cell multiomic sequencing on epithelium-enriched cells from another naive HGSOC patient tumor. We also utilized the Adaptively Thresholded Low-Rank Approximation imputation approach to eliminate technical zeroes while preserving biological zeroes (~15%–40% of genes) in the expression data (48). Using these optimized experimental and analytic tools, we confirmed that most of the captured cells were epithelial cells by examining EPCAM expression (Figure 4D). Among the 7,273 epithelial cells, we identified a small population of cells (*n* = 46 total) that were significantly enriched for *SOX9* expression relative to the other clusters (Figure 4, E–G). This validated our revised approach, as a cluster of this size was undetectable in the unenriched sample (Figure 4, B and C).

### SOX9-expressing epithelial cells are enriched for CSC properties.

We used multimodal scRNA- and scATAC-Seq to cluster the cells based on both modalities using WNN clustering (Figure 4, A, B, D, and E) (49). This technique’s power is derived from its ability to strengthen the characterization of the identified clusters. Using this clustering technique, we identified clusters at the chromatin accessibility level while simultaneously preserving the clustering at the transcriptional level (Figure 4, F, H, and I, and Supplemental Figure 3). Since cluster 11 in the epithelium-enriched, scRNA-Seq–imputed patient sample was significantly enriched for *SOX9* expression (highest Wilcoxon’s FDR = 2.97e-06) (Figure 4F), we sought to better understand what type of cell it represented. We first looked at the expression of a stress response gene signature (*n* = 35 genes) identified by Zhang et al. in the longitudinal HGSOC scRNA-Seq dataset (Figure 2) (40). This gene set was found to be enriched in chemo-treated patient samples and was shown to promote platinum resistance in OC through the induction of proinflammatory responses, epithelial-mesenchymal transition (EMT), prosurvival programs, and stemness. This gene set is an extremely poor prognostic indicator in HGSOC patients. In our data, cluster 11 — the only cluster enriched for SOX9 expression — was also significantly enriched for the stress response gene signature (highest Wilcoxon’s FDR = 2.42e-07) compared with any other cell cluster (Figure 4H). Next, we investigated the expression of canonical ovarian CSC markers that are commonly used to identify CSCs. We created a gene module of 12 OC CSC markers (PROM1, CD44, ALDH1A1, CD24, KIT, ENG, VCAM1, EPCAM, NES, SOX2, SSEA1, and THY1) and plotted their expression across every cluster in the dataset (Figure 4I) (50). We confirmed that cluster 11 was the most significantly enriched for this signature (highest Wilcoxon’s FDR = 3.8e-15) compared with any other cell cluster. An Enrichr Gene Ontology (GO) term analysis (51) of the differentially expressed genes in cluster 11 (GO Biological Process database; Benjamini-Hochberg FDR < 0.005, log2FC > 0, *n* = 311) showed an enrichment for antiapoptotic processes, negative regulation of growth, regulation of stem cell maintenance, and negative regulation of differentiation (Figure 4J) (52). These analyses further indicate that SOX9-expressing cells exhibit CSC properties and that SOX9 is a potential regulator of this population. Next, we analyzed the ATAC-Seq peaks in this cluster to identify TFs that might be selectively associated with the open chromatin regions. To this end, we performed motif enrichment analysis on the scATAC fragments using ChromVAR (Figure 4K) (53) and found that the most enriched motifs for cluster 11 are almost all SOX family TFs, including SOX9. Lastly, we used the GREAT annotation tool to find GO term enrichment of the accessible regions in cluster 11 (Figure 4L) (54). This analysis showed that these cells activate stress-related pathways such as unfolded protein response, mitochondrial outer membrane permeabilization, proteasome assembly, and G1 DNA damage checkpoint. They are also enriched for pathways vital for cell growth and proliferation, such as telomere maintenance and JUN kinase activation, further affirming SOX9’s association with stress response and growth.

### SOX9 expression is sufficient to drive chemoresistance in HGSOC cells.

**Figure 5:**
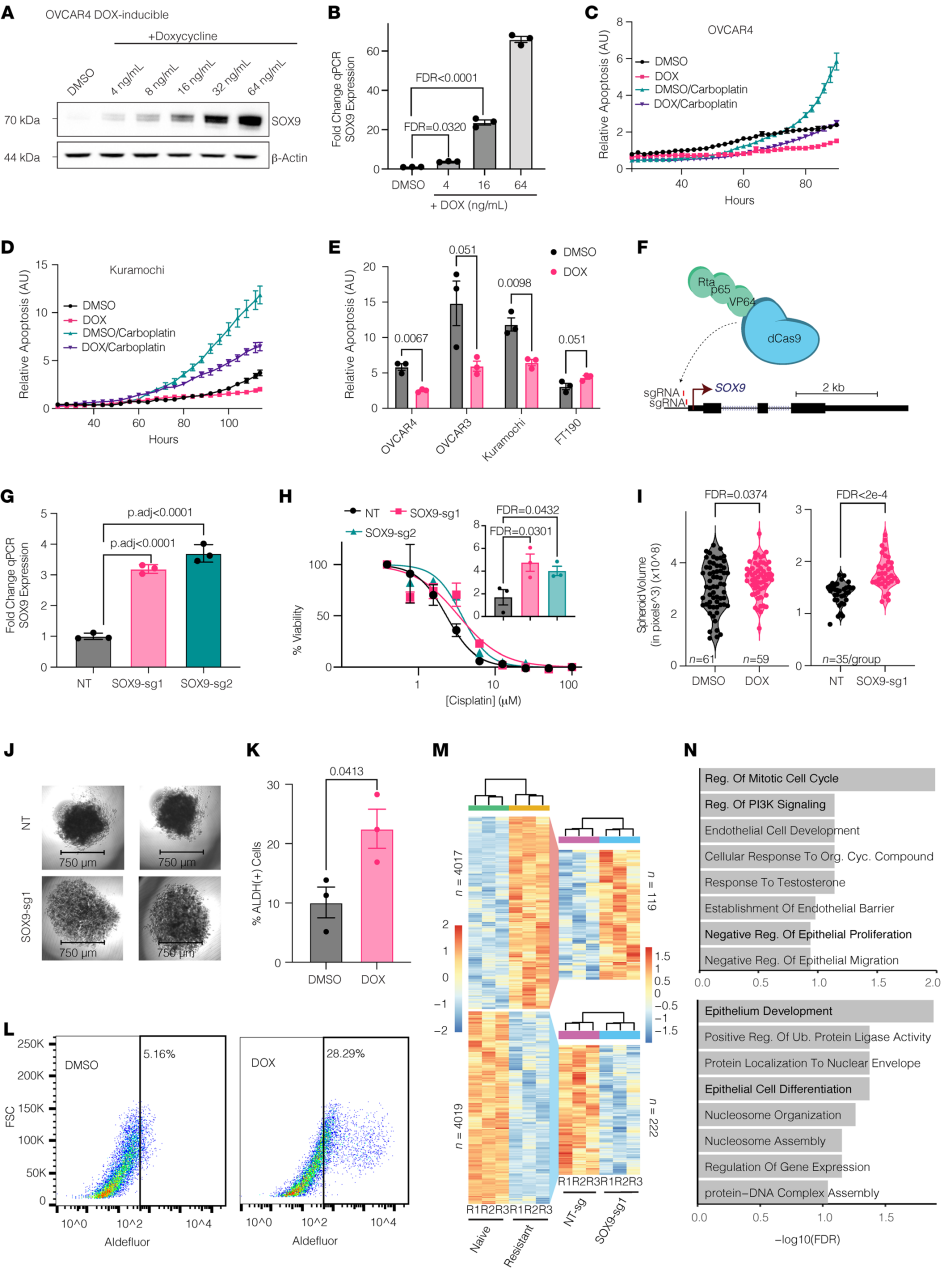
Ectopic SOX9 induction increases platinum resistance and stemness in cell line models. (**A**) Western blot showing SOX9 in OVCAR4-SOX9OE DOX-inducible cells (4–64 ng/mL DOX). (**B**) Normalized qPCR SOX9 expression in OVCAR4-SOX9OE cells with increasing DOX. Mean ± SEM, *n* = 3. Significance by 1-way ANOVA with 2-stage step-up Benjamini, Krieger, and Yekutieli correction for multiple comparisons. (**C**–**E**) IncuCyte measurements of normalized apoptosis of OVCAR-, Kuramochi-, OVCAR3-, and FT190-SOX9OE cells treated with DMSO or DOX and carboplatin. Mean ± SEM, *n* = 3. Significance by multiple 2-tailed paired Student’s *t* tests with Benjamini, Krieger, and Yekutieli correction for multiple comparisons. (**F**) Overview of the OVCAR4-VPR system. (**G**) Normalized qPCR SOX9 expression in OVCAR4-VPR cells with nontargeting (NT) or SOX9-targeting sgRNA. Mean ± SEM, *n* = 3. Significance by 1-way ANOVA with 2-stage step-up Benjamini, Krieger, and Yekutieli correction for multiple comparisons. (**H**) MTT cell viability assay of OVCAR4-VPR cells with same sgRNA as in **E** and increasing cisplatin (0–100 µM) and IC_50_ values (inset). Mean ± SEM, *n* = 3. Significance by 1-way ANOVA with 2-stage step-up Benjamini, Krieger, and Yekutieli correction for multiple comparisons. (**I**) Spheroid volumes (in pixels^3^) for OVCAR4-VPR system and OVCAR4-SOX9OE DOX-inducible system. FDR by 2-tailed Student’s *t* test. (**J**) Images of OVCAR4-SOX9OE spheroids treated with 0.1 µg/mL DOX or equivalent volume of DMSO. (**K**) Percent of OVCAR4-SOX9OE DOX-inducible cells from spheroids that were found to be ALDH(+) using an Aldefluour kit. *P* value by 2-tailed Student’s *t* test. *n* = 3. (**L**) Example FACS profiles of two data points in **K**. (**M**) Differentially expressed genes (*P* < 0.05) from RNA-seq of OVCAR4-VPR cells with NT- or SOX9-sg1 and OVCAR4 platinum-naïve/resistant cells. (**N**) GO term analyses of commonly upregulated (top) and downregulated (bottom) genes between OVCAR4 platinum-resistant versus naïve and OVCAR4-VPR + SOX9sg1 versus NT-sg cells. Shown are odds ratios and –log_10_(FDR).

Our data so far support the hypothesis that SOX9 is associated with and potentially drives HGSOC chemoresistance. To experimentally test the sufficiency of SOX9 for chemoresistance, we transduced platinum-naive OVCAR4, Kuramochi, OVCAR3, and FT190 immortalized FTE cells with a doxycycline-inducible (DOX-inducible) SOX9 construct under the control of tetracycline-responsive elements (55). With the expression of reverse-tTA effector pUltra-puro-RTTA3, we titrated the induced SOX9 protein levels (Figure 5, A and B). SOX9 induction in OVCAR4, Kuramochi, and to a lesser extent OVCAR3 cell lines lead to increased platinum resistance, as measured by an Incucyte long-term live-cell imaging platform (Figure 5, C–E, and Supplemental Figure 4). Interestingly, the FT190 cells displayed no such advantage, suggesting that SOX9 only has this effect in a cancer cell context (Figure 5E). We assessed cell proliferation and apoptosis rates using activated caspase-3/7 dye normalized to phase confluence. This was necessary because we found that with increased SOX9 expression, cell proliferation slowed significantly (Supplemental Figure 5). This finding agrees with our GO term analysis in the multimodal single-cell dataset that showed an enrichment of genes involved in the negative regulation of cell growth. In the absence of carboplatin, both DOX- and DMSO-treated cells exhibited a low basal level of apoptosis. However, when treated with carboplatin, the DOX-treated SOX9-overexpressing cells exhibited significantly lower relative apoptosis compared with the control cells (OVCAR4 FDR = 0.0067, OVCAR3 FDR = 0.051, and Kuramochi FDR = 0.0.0098), indicating that SOX9 induces platinum resistance in OC cell lines.

### CRISPR-mediated epigenetic activation of endogenous SOX9 drives chemoresistance in HGSOC cells.

Encouraged by the SOX9 induction results, we investigated whether physiological levels of SOX9 could also result in chemoresistance. We observed that chemoresistant OC cells have approximately 3-fold higher SOX9 protein levels compared with naive cells (Figure 1C). Despite enabling dose-dependent protein expression, it is challenging to achieve such a low level of protein induction in a DOX-inducible system. When characterizing TFs, overexpression can have nonphysiological impacts on downstream regulatory elements, particularly in the case of SOX9 (56). We therefore utilized a CRISPR epigenetic activation approach in which targeted recruitment of a dCas9-fused VPR (VP64-p65-Rta) (57) transactivation domain epigenetically activated endogenous loci through the recruitment of general transcriptional machinery. Targeting *SOX9*’s promoter with 2 sgRNAs in OVCAR4 cells enabled us to increase the endogenous *SOX9* expression by 3- to 4-fold (Figure 5, F and G). These are comparable levels to those we observed in cells following platinum treatment (Figure 1, C and D). These cells were significantly more resistant to platinum treatment, as measured by an MTT cell viability assay (Figure 5H). We found that the half maximal inhibitory concentration (IC50) of cisplatin was increased around 2-fold, confirming that physiologically relevant SOX9 expression is sufficient to drive chemoresistance.

### Epigenetic activation of endogenous SOX9 results in stem-like cellular phenotypes and transcriptional states.

To directly test the stem-inducing capability of SOX9, we utilized a hanging droplet spheroid formation assay as a proxy for stemness. In both the DOX-inducible and epigenetic-mediated SOX9 induction OVCAR4 cells, we found that the spheroids that formed were significantly larger in the SOX9-induced cells (Figure 5, I and J). To further confirm this stemness phenotype, we used an Aldefluor kit on dissociated spheroids to directly measure aldehyde dehydrogenase (ALDH) activity, an established marker of OC stem cells (58). Flow cytometry showed that SOX9 induction results in a population of cells with significantly higher ALDH activity, suggesting formation of CSCs (Figure 5, K and L). In line with these data, OVCAR3 cells also produced significantly larger spheroids when induced to express SOX9 (Supplemental Figure 6). Importantly, induced SOX9 expression in noncancerous FT190 FTE cells also increased overall stemness. Using the Aldefluor kit, we found that FT190 spheroids also form a distinct stem cell population upon SOX9 induction. Interestingly, while these spheroids were not larger, multiple spheroids formed within each droplet when SOX9 was induced (Supplemental Figure 7). Taken together, these data show that SOX9 induces a CSC-like population in HGSOC cells and that it might be involved in normal stem maintenance in the FTE.

To reveal transcriptional mechanisms by which SOX9 drives chemoresistance, we performed RNA-Seq on both OVCAR4 naive/platinum-resistant cell pairs and dCas9-VPR–expressing naive cells expressing control and *SOX9* promoter–targeting sgRNA (Figure 5M). We identified 4,017 upregulated and 4,019 downregulated genes in OVCAR4 platinum-resistant cells compared with naive cells (Wilcoxon’s *P* < 0.05). We then looked at which of these 8,036 genes were also differentially expressed between the control and *SOX9* sgRNA cell lines (*P* < 0.05). We found that 119 genes were commonly upregulated, while 222 genes were commonly downregulated between these groups. The Enrichr analysis of the top represented GO Biological Process terms indicated that commonly upregulated genes were enriched for phosphatidylinositol 3-kinase signaling and negative regulation of epithelial cell proliferation, while the downregulated genes were enriched for epithelial cell differentiation (Figure 5N). These findings further support the hypothesis that SOX9 expression enhances stemness (59, 60) and blocks epithelial differentiation and proliferation.

### Acute cisplatin treatment uniformly induces SOX9 expression and stemness pathways at the single-cell level.

**Figure 6:**
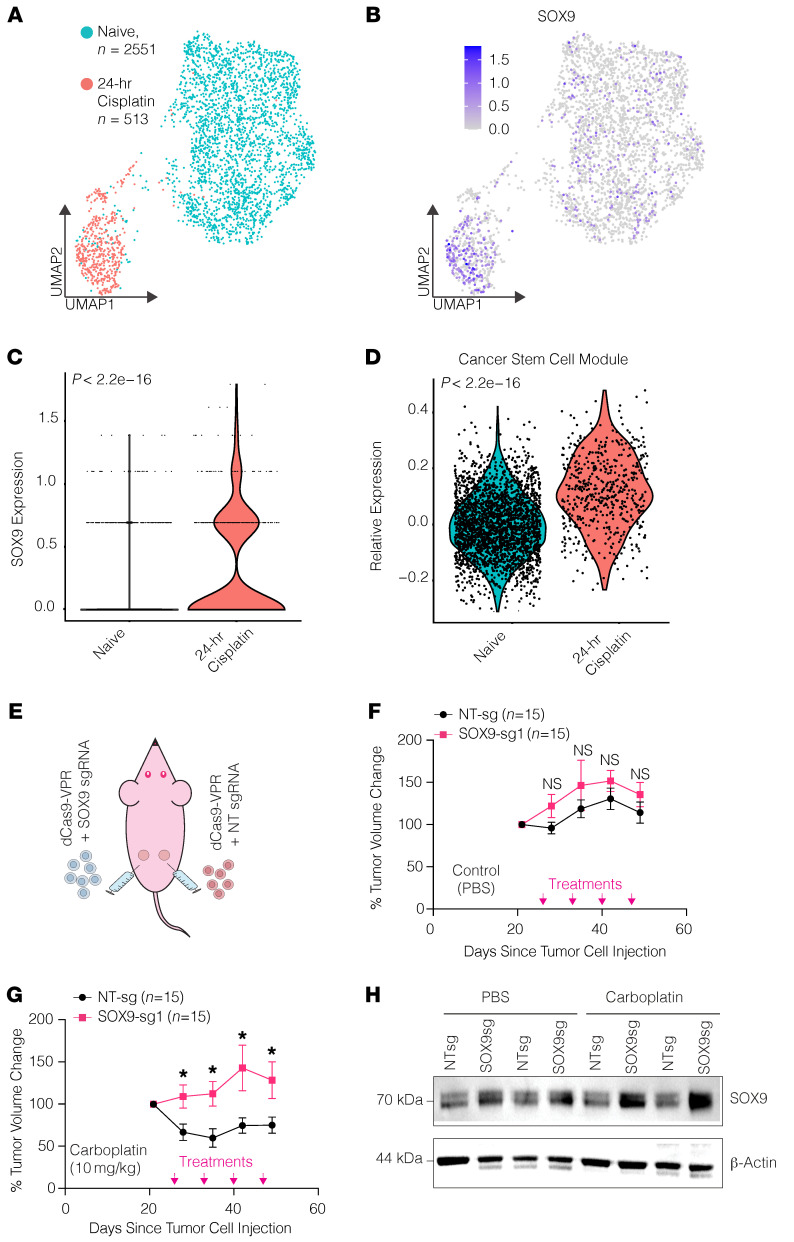
Acute platinum treatment induces both SOX9 and OC stem genes in OVCAR4 cells regardless of CSC status, and SOX9 increases platinum resistance in vivo. (**A**) UMAP plot showing scRNA-Seq clustering for both cisplatin-treated and untreated OVCAR4 cells. (**B**) UMAP feature plot showing *SOX9* expression distribution in cisplatin-treated and untreated OVCAR4 cells. (**C**) Violin plot showing *SOX9* expression at a single-cell level in cisplatin-treated and untreated OVCAR4 cells. *P* value was calculated using Wilcoxon’s signed-rank test. (**D**) Violin plot showing OC stem module expression at a single-cell level in cisplatin-treated and untreated OVCAR4 cells. *P* value was calculated using Wilcoxon’s signed-rank test. (**E**) Depiction of in vivo study design and results. Thirty nude mice had 2 × 10^6^ OVCAR4-VPR cells (50% Matrigel by volume) injected subcutaneously in either flank: one side with NT sgRNA and the other with SOX9 sgRNA. The mice were then treated either with 10 mg/kg carboplatin or the equivalent volume of PBS 4 times, and tumor volume was measured once a week. (**F**) Percent change in tumor volume starting 1 week before treatment in the PBS-treated (*n* = 15) group. Significance was calculated using multiple 2-tailed paired Student’s *t* tests with Benjamini, Krieger, and Yekutieli correction for multiple comparisons. From left to right, FDR = 0.2147, 0.3867, 0.2147, and 0.3256. (**G**) Percent change in tumor volume starting 1 week before treatment in the 10 mg/kg carboplatin (*n* = 15) group. Significance was calculated using multiple 2-tailed paired Student’s *t* tests with Benjamini, Krieger, and Yekutieli correction for multiple comparisons. From left to right, FDR = 0.0231, 0.0231, 0.0324, and 0.0324. (**H**) Western blot depicting mouse-paired tumor SOX9 levels across treatments.

The single-cell data from the longitudinal study and the immune staining in TMAs indicated that SOX9 expression is uniformly upregulated after chemotherapy. However, these experiments could not determine whether the post-NACT cells are originally derived from a rare population of SOX9-expressing cells. To better understand this and the early steps of chemoresistance, we performed scRNA-Seq on naive OVCAR4 cells that were control or cisplatin treated for 24 hours (Figure 6, A–D). In line with our previous findings, the dimensional reduction of the data shows that the transcriptional landscape is drastically altered when the cells are acutely treated with cisplatin (Figure 6A). More critically, we observed a significant increase in SOX9 expression and in the number of cells with detectable *SOX9* mRNA following acute cisplatin treatment (Figure 6, B and C). Additionally, we saw a significant and uniform increase in the same 12 OC stem markers that we had previously found only in a small CSC population in chemo-naive tissue (Figure 6D). These findings show that in a cell line model, both SOX9 and canonical stem markers are acutely and uniformly upregulated following acute platinum exposure, indicating that chemoresistance might begin with global transcriptional reprogramming at the population level and that SOX9 is a critical regulator of this process.

### Epigenetic upregulation of endogenous SOX9 induces platinum resistance in vivo.

Based on these in vitro experimental results, we next tested whether the chemoresistance phenotype would be recapitulated in tumors in vivo. To this end, we subcutaneously (both flanks) injected OVCAR4-VPR cells expressing nontargeting (NT) sgRNA (right flank) or SOX9 promoter–targeting sgRNA (SOX9-sg1, left flank). After tumors were established for 3 weeks, the mice were treated weekly with either PBS (vehicle) or 10 mg/kg carboplatin (Figure 6E) and the size of tumors was monitored. We found that, within just 2 treatment periods, the tumors expressing the NT sgRNA had decreased in size significantly compared with the tumors expressing the SOX9 promoter-targeting sgRNA (Figures 6, E and F, and Supplemental Figures 8 and 9). Upregulation of SOX9 was confirmed by Western blotting in protein lysates collected from mouse-matched tumors (Figure 6H). These data recapitulated our in vitro results and further demonstrated the clinical relevance of our findings that SOX9 expression is a critical driver of chemoresistance in HGSOC.

## Discussion

The largest hurdle in the treatment of HGSOC is the high frequency of recurrence following the administration of neo-adjuvant platinum treatments. HGSOC cells develop chemoresistance through diverse molecular mechanisms that are driven by transcriptional and epigenetic reprogramming (3, 61). There are 2 plausible models of chemoresistance: naive tumors contain inherently resistant stem-like cells, or all cells are capable of globally reprogramming their transcriptome after chemotherapy treatment to gain a stem-like state (9). However, these competing models are not necessarily mutually exclusive. It is possible, for instance, that a small heterogeneous population of naive cells responds to chemotherapy by stochastically inducing a small number of master regulators that can in turn create the malleability required for chemoresistance. Aside from the route to chemoresistance and acquisition of a stem-like state, our knowledge of the key drivers of this state is limited. In this study, we identified SOX9 as a key TF that governs chemoresistance and the transcriptional program of OC stem cells.

Through the analyses of large-scale TMAs and scRNA-Seq datasets in primary patient samples, we established that SOX9 expression and activity are significantly increased in most HGSOC tumors following NACT. The relative uniformity of SOX9’s expression in these cells supports the hypothesis that the majority of the cells in this tissue are potentially capable of reprogramming their transcriptional state to gain a stem-like phenotypic response to treatment. The analysis of a single-cell expression dataset of naive and 24-hour acute cisplatin-treated cells further supported this hypothesis. Importantly, OVCAR4 cells are estimated to double in around 43 hours (62). The fact that we observed significant transcriptional reprogramming of SOX9 loci and other stem cell genes within the first 24 hours of chemo treatment indicates that the observed global transcriptional reprogramming is not due solely to the survival/proliferation of inherently resistant stem-like cells. In fact, our live-cell imaging experiments showed that increased expression of SOX9 significantly slows the proliferation of these cells (Supplemental Figure 5), while SOX9-depleted cells proliferate faster (Supplemental Figure 1). These findings suggest a potential mechanism whereby slow cycling, high SOX9-expressing cells have a higher tendency to tolerate DNA-damaging chemotherapy because they have reduced DNA replication rates while also upregulating the expression of DNA damage repair and unfolded protein response pathway genes.

Notably, SOX9 was one of the top TFs significantly correlated with transcriptional divergence in primary HGSOC cells. Transcriptional divergence is a proxy for transcriptional malleability, a key hallmark of CSCs. Consistent with this, the only TF that ranked above SOX9 for this measurement, NR4A1, is involved with both chromatin structure and global transcriptional changes (63). It is also important to note that SOX9 is associated with transcriptional divergence more significantly than even the known oncogenic stem factors, such as MYC and KLF4, further emphasizing its role in inducing a stem-like transcriptional state.

Further support for this hypothesis came from the identification of a rare, small, stem-like cancer cell population in our multimodal single-cell dataset of chemo-naive tumors. To our knowledge, this is the first time such a rare population has been directly identified through single-cell profiling of primary HGSOC naive tumors. We found that SOX9 is a key marker of this rare cell population both at the expression level and from the motif analysis of differentially accessible chromatin sites (ATAC-Seq peaks) specific to this population. These findings indicated the presence of a rare cluster of cells in naive tumors that demonstrate a stem-like transcriptional state defined by high SOX9 expression and its motif accessibility. The necessity of SOX9 for transcriptional plasticity has previously been seen in breast (64), lung (65), and pancreatic cancers (66); however, it has been unclear whether SOX9 is merely a marker of plasticity or if it can induce these stem properties in non-CSCs to survive cytotoxic chemotherapy. Previous studies have shown SOX9 expression linked to hypoxic response through HIF-2α, BCL-2, and KI-67 in sex cord stromal OC (a rare OC subtype) (67, 68). Identified associations with stemness, however, have been largely relegated to surface markers such as CD44, PROM1, and CD117 (69). Through CRISPR-based, locus-specific, epigenetic activation (dCas9-VPR), we demonstrated that inducing endogenous SOX9 expression significantly increases platinum resistance in OVCAR4 cells and that this expression also induces stem-like transcriptional programs. The benefits of activating the endogenous loci compared with a traditional overexpression approach are 2-fold. First, the induced expression is limited by endogenous copies of the gene. Second, the negative control for the system consists of a NT sgRNA, thus avoiding the confounding issues of chemical treatment and leaky expression.

Most compellingly, we found that induction of SOX9 either exogenously or endogenously increases the capacity of OVCAR4 and OVCAR3 cells to form spheroids and induce the formation of a distinct ALDH^+^ CSC population in OVCAR4 cells. Interestingly, we found that certain FTE tissues within our TMAs contain relatively high SOX9 staining (Figure 3C). Moreover, when induced to express exogenous SOX9, normal FT190 cells formed larger spheroids and a distinct ALDH^+^ stem cell population (Supplemental Figure 7). Previous studies have shown that there is an enrichment of stem-like epithelial cells in the distal (fimbrial) ends of the FTE (70), as these regions are more prone to injury and ROS due to exposure to follicular fluid released during ovulation. As a result, it is believed that the distal FTE is a likely HGSOC tissue of origin. Our finding that SOX9 induces the formation of stem populations in FTE cells warrants further studies to test the hypothesis that SOX9 upregulation might be the critical factor that mediates the transformation and initiation of cells that result in HGSOC.

Overall, our findings indicate that SOX9 is not just a key marker of stemness and platinum resistance in HGSOC, but is also sufficient to induce these phenotypes. We found evidence that, upon acute exposure to platinum, nearly all cancer cells upregulate SOX9, thereby inducing overall stem state–relevant genes. This indicates that acute treatment increases overall transcriptional divergence and malleability at the population level. Through this increased malleability, these cells can then enhance stress-related pathways vital to chemoresistance, such as G1/S checkpoint, growth suppression, and unfolded protein response, while simultaneously inhibiting differentiation. Importantly, from a clinical standpoint, these findings suggest that targeting CSCs in treatment-naive tissues might not be sufficient to prevent recurrence in these cancers as the remaining cells could still have the capacity to reprogram their transcriptional state. Therefore, identifying and targeting key drivers of this reprogramming, such as SOX9, might be a more relevant therapeutic route for understanding and preventing chemoresistance.

## Methods

### Sex as a biological variable.

Because this study was focused only on effects in OC, sex was not a variable in any of the experiments. As such, all cell lines and animals used were female.

### Cell culture.

Human OC cell lines OVCAR4 and Kuramochi were cultured in complete medium consisting of RPMI 1640 (Thermo Fisher Scientific; catalog 11875093), 10% heat-inactivated FBS (Hyclone; catalog SH30071.02HI), and 1% penicillin/streptomycin (Pen/Strep; Thermo Fisher Scientific; catalog 15070063). Human OC cell line COV362 was cultured in complete medium consisting of DMEM (Thermo Fisher Scientific; catalog 11965092), 10% heat-inactivated FBS, 1% Pen/Strep, and 1× Glutamax (Thermo Fisher Scientific; catalog 35050061). Human OC cell line OVCAR3 was cultured in complete medium consisting of RPMI 1640, 20% heat-inactivated FBS, 1% Pen/Strep, and 12.8 μg/mL human recombinant insulin (1.6 mL of 4 mg/mL stock; Thermo Fisher Scientific; catalog 12585-014). Human embryonic kidney cell line HEK293T was cultured in complete medium consisting of DMEM, 10% FBS, and 1% Pen/Strep. The Kuramochi and COV362 cell lines were provided in-house, the OVCAR4 and OVCAR3 cell lines were obtained from Charles Landen (University of Virginia, Charlottesville, Virginia), and the HEK293T cell line was obtained from ATCC. The cells were grown in an incubator at 37°C in a humidified atmosphere containing 5% CO_2_ and 95% air. The cells were validated using the ATCC STR profiling service and were periodically assayed for mycoplasma contamination.

### Creating the platinum-resistant ovarian cell line.

OVCAR4 platinum-resistant cells were created as previously described (18). Briefly, cells were treated with an initial dose of 1 μM cisplatin for 4 hours before being washed with PBS and allowed to recover in normal full medium for 2 passages. This was repeated until the cells showed a significant increase in their platinum sensitivity, as measured by MTT assay.

### Creating SOX9-overexpressing and SOX9-KO cell lines.

Plasmids FUW-tetO-SOX9 (Addgene 41080), pUltra-puro-RTTA3 (Addgene 58750), lenti-EF1a-dCas9-VPR-Puro (Addgene 99373), CROP-seq-Guide-mCherry (modified from Addgene 86708 to have mCherry instead of PuroR), and lentiCRISPR v2 (modified from Addgene 52961 to have a BsmBI Goldengate Cloning sgRNA insertion site) were obtained from Addgene. To create lentivirus, HEK293T cells were seeded into a 10 cm^2^ treated plate at a density of 4 × 10^6^ cells/plate. The following day, the cells were transfected using 4 μg of transgene plasmid, 2 μg of psPAX2 (Addgene 12260), 1 μg pMD2.G (Addgene 12259), and 21 μg of polyethylenimine (PEI MAX; 45,000 MW; Polysciences; catalog 24765-1) in Opti-MEM reduced serum medium (Thermo Fisher Scientific; catalog 31985070) that had been incubated at room temperature for 15 minutes before being added to the cell medium dropwise. The cells were washed with PBS, and the cell medium was replaced the next day. After 48 hours, the cell medium was removed, filtered using a 0.45 μm syringe filter (Corning; catalog 431220), aliquoted, and stored at –80°C.

For infection, cells were seeded at a density of 2 × 10^5^ cells per well into a 6-well treated plate. The next day, the cell medium was removed and replaced with 1 mL of the appropriate viral medium along with 10 μg/mL polybrene (MilliporeSigma; catalog TR-1003-G). One day later, the viral medium was removed, the cells were washed, and normal medium was added. For cells transduced with the pUltra-puro-RTTA3, the SP-dCas9-VPR transgenes, or the lentiCRISPR v2 SOX9-KO construct, 24 hours later, cells were selected with 1 μg/mL puromycin (InvivoGen; catalog ant-pr) until all the cells in a negative control (untransduced) well were dead. These cells were subsequently transduced a second time with the FUW-tetO-SOX9 or CROP-seq-Guide-mCherry viral medium and either immediately used in downstream protocols or enriched using FACS, respectively. Guide sequence oligos used were as follows: SOX9-sg1, GGGAGGGGATCGCAGCCAAA; SOX9-sg2, GCAGCCAAAGGGCGGACGGT; NT-sg, CACCGAACTCAACCAGAGGGCCAA; and SOX9KO-sgRNA, CACCACGTCGCGGAAGTCGATAGG.

### Colony formation assay.

OVCAR4 cells with either a luciferase-targeting (NT) or SOX9-targeting (SOX9-KO) sgRNA and CRISPR/Cas9 were seeded into a 12-well plate at a density of 500 cells/well. The following day, half of the wells were treated with 20 μM carboplatin. After 24 hours, the carboplatin was removed and replaced with complete medium. One week later, the colonies were visualized using 0.5% crystal violet (Fisher Scientific; catalog C581-100) in 20% methanol. Briefly, the medium was carefully removed and replaced with 0.5 mL of crystal violet solution. The plate was wrapped in aluminum foil and allowed to incubate on a shaker at room temperature for 1 hour. Next, the dye was removed, and the plate was washed 3 times with PBS before being allowed to dry in the dark overnight. After scanning the plate wells, we used the ColonyArea ImageJ (NIH) plug-in to determine the percentage of colony coverage per well (71).

### Incucyte live-cell analysis and apoptosis assay.

Cells were seeded in their respective medium into 96-well plates in triplicate at a density of 1,000 cells per well. The following day, the medium was replaced with fresh complete medium or complete medium containing either 64–100 ng/mL DOX (Sigma-Aldrich; catalog D9891-1G; 64 ng/mL for OVCAR4 and 100 ng/mL for all other cell lines) or the same volume of DMSO (Fisher Scientific; catalog D128-1). After 24 hours, the medium was again removed and replaced with fresh medium with the same treatments along with 1:1,000 Incucyte caspase-3/7 dye (Sartorius; catalog 4440) and carboplatin (50 μM for OVCAR4, 10 μM for OVCAR3, 5 μM for Kuramochi, and 10 μM for FT190). The plate was then incubated in an Incucyte live-cell imager that was programmed to capture 4 images per well in phase and green every 2 hours for approximately 4 days. Green mean integrated intensity (green calibrated unit [GCU]/mm^2^) and phase confluence (%) were exported and loaded into GraphPad Prism version 10 (Dotmatics) to plot. Apoptotic cells were next normalized to total cell coverage to plot the relative amount of apoptotic cells per sample.

### MTT cell viability assay.

Cells were seeded in their respective medium into a 96-well plate in triplicate at a density of 5,000 cells per well. The following day, the medium was replaced with complete medium containing a 1:1 serial dilution of cisplatin (from 100 to 0.39 μM) with the last wells receiving no treatment as negative control. The cells were incubated with the treatment for 24 hours, at which point the medium was removed and the cells were washed with 100 μL of PBS (Thermo Fisher Scientific; catalog 14190094) before being replaced with complete medium. After 48 hours, the medium was removed and replaced with 90% fresh medium along with 10% of a stock solution of MTT (Invitrogen; catalog M6494) at 5 mg/mL in PBS. The cells were then returned to the incubator for an additional 4 hours before an equal volume of MTT solubilization buffer (10% SDS and 0.1% Tris HCl, pH 8) was added. The plate was allowed to incubate an additional 10 minutes, covered in foil and with rocking, before being read on a plate reader at 590 nm. Background absorbance was measured as the readings for control wells that contained no cells. Absorbance was normalized to the lowest treatment concentration, data were plotted, and IC_50_ was measured using GraphPad Prism version 10.

### Hanging droplet and Aldefluor assays.

Cells were lifted, counted, and diluted to a concentration of 0.5 × 10^6^ cells per mL. If the cells were DOX inducible, they were diluted in either 100 ng/mL DOX or the same volume of DMSO as a control. Next, 5 mL of PBS was added to a 6 cm plate before the lid was turned over, and 20 μL droplets of the diluted cells were added. Up to 20 droplets were added per plate. The lids were then carefully flipped back onto the plate before the spheroids were allowed to grow for 7 days. At the end of the 7 days, cell images were taken using a ×4 lens, or the cells were prepared for the Aldefluor assay. Spheroid images were analyzed using Adobe Photoshop’s object selection tool, and volume was calculated using the area and diameter of the spheroids. Spheroids used for the Aldefluor assay were pelleted at 1,000*g* for 5 minutes and resuspended in 1 mL of TrypLE Select Enzyme (1×) (Thermo Fisher Scientific; catalog 12563011). The pelleted spheroids were allowed to digest for 5 minutes at 37°C before being pelleted again and resuspended in full medium to quench the enzyme. The Aldefluor samples were prepared using the Aldefluor kit (STEMCELL Technologies; catalog 01700) according to the manufacturer’s specifications, and ALDH activity was measured via FACS on a BD FACSMelody cell sorter (BD Biosciences). FACS analysis was performed using FlowJo version 10.10.0 (BD Biosciences).

### Western blot.

Cell lysates were collected with 1× RIPA buffer (Boston BioProducts; catalog BP-115) with protease inhibitor cocktail tablet (Thermo Fisher Scientific; catalog A32953; 1 tablet/10 mL buffer). The lysates were quantified using a BCA Protein Assay Kit (Thermo Fisher Scientific; catalog A55864). The lysates were run on a NuPAGE 4%–12% Bis-Tris 1.0–1.5 mm protein gel (Thermo Fisher Scientific; catalog NP0321) and transferred to a nitrocellulose membrane using the iBlot dry blotting system (Thermo Fisher Scientific; catalog IB1001) at 20 V for 7 minutes. The membranes were blocked with 3% nonfat dry milk (Bio-Rad; catalog 170-6404). The SOX9 antibody was diluted to 1:1,000 (Abcam; catalog Ab182579), and the β-actin antibody was diluted to 1:10,000 (Sigma-Aldrich; catalog A1978). Anti-mouse and anti-rabbit IgG HRP-conjugated secondary antibodies were used, diluted to 1:2,500 or 1:10,000 for loading control (Promega; catalog W402B and W401B, respectively). Membranes were visualized in an iBright FL1500 imaging system (Thermo Fisher Scientific; catalog A44241) using either ECL substrate (Cytiva; catalog GERPN2209) or SuperSignal West Femto maximum sensitivity substrate (Thermo Fisher Scientific; catalog 34096).

### Reverse transcription quantitative PCR gene expression analysis.

RNA was extracted from cells directly on a 6-well plate using a Quick-RNA MiniPrep kit (Zymo Research; catalog R1054) according to the manufacturer’s instructions. cDNA was constructed using a High-Capacity cDNA Reverse Transcription Kit (Thermo Fisher Scientific; catalog 4368814) using the on-column gDNA digestion step according to the manufacturer’s instructions. Reverse transcription quantitative PCR (RT-qPCR) was performed on a QuantStudio 3 (Thermo Fisher Scientific; catalog A28567) using 3 μL of 1:200 diluted cDNA, 4.5 μL of 10 μM forward (F)/reverse (R) primers in water, and 7.5 μL SYBR green 2× Master Mix (Applied Biosystems; catalog 4385612) for a total 15 μL run for 40 cycles plus melt curve. Primers used were as follows: SOX9-F, GGCAAGC CTCTGGAGACTTCTG; SOX9-R, CCCGTTCTTCACCGACTTCC; β-actin-F, AGAGCTACGAGCTGCCTGAC; and β-actin-R, AGCACTGTGTTGGCGTACAG.

### TMA staining and analysis.

TMA staining was performed using the SOX9 antibody (MilliporeSigma; catalog 5535) according to the manufacturer’s recommendations.

TMA images were analyzed using QuPath software (46). Fourteen regions of tissues across both slides, as well as patient and tissue type, were manually annotated as either stromal or epithelial according to physical morphology. QuPath’s machine learning algorithm was then trained on these annotations and allowed to automatically annotate the remaining tissue samples. The h-scores were calculated by first taking the nuclear DAB staining means per cell and finding the number of cells per tissue that fall into the low, medium, or high quantile of all cells across all tissues. Next, for each individual tissue, these numbers (low_n, medium_n, and high_n) were plugged into the following equation: h = 100 * ((3*high_n + 2*medium_n + 1*low_n)/(high_n + medium_n + low_n + total_n)).

### scRNA-Seq analysis.

The preintegrated and quality-checked count matrix and metadata table were downloaded from the Gene Expression Omnibus (GEO) (accession code GSE165897). The counts and metadata were analyzed in R using Seurat version 4.4.0 (49). Briefly, raw counts were transformed and normalized using SCTransform before principal component analysis (PCA) was performed. Next, using 20 dimensions, Uniform Manifold Approximation and Projection (UMAP) was used to plot the data followed by clustering. Epithelial cells had previously been identified within the metadata, so after filtering for only epithelial cells, PCA, UMAP, and clustering were reperformed. Transcriptional divergence was calculated at a single-cell level by first removing all nonexpressed genes and then finding the ratio between the count sum of the top 50% of expressed genes versus the count sum of the bottom 50% as previously described (44).

### Multimodal single-cell tissue and library preparation.

The fresh tissues were washed with cold PBS and mechanically disrupted to generate small pieces. Then, they were subjected to enzymatic digestion with a tissue dissociation kit (Miltenyi Biotec; catalog 130-095-929) following the manufacturer’s instructions. Enzymatic digestion was carried out for 2 hours at 37°C with shaking at 0.5*g*. The digested samples were then filtered through 70 μm cell strainers and subjected to red blood cell lysis using RBC lysis buffer (eBioScience; catalog 00-4333-57). Cells were counted and subjected to tumor cell enrichment for the samples indicated.

For the tumor cell enrichment, we used a tumor cell isolation kit (Miltenyi Biotec; catalog 130-108-339) following the manufacturer’s instructions. Briefly, the samples were incubated with a cocktail that magnetically labeled non–tumor cells and passed through LS columns (Miltenyi Biotec; catalog 130-042-401) attached to a MACS multistand (Miltenyi Biotec; catalog 130-042-303). Tumor cells were eluted and collected, and the cell number and viability were analyzed.

Enriched tumor cells or cells isolated from the tissues were then subjected to DNase treatment and nuclei isolation following the Nuclei Isolation for Single Cell Multiome ATAC + Gene Expression Sequencing protocol by 10X Genomics (CG000365 rev. B). A total of 10,000 nuclei were targeted per sample.

The isolated nuclei were immediately used for transposition, followed by gel bead-in-emulsion (GEM) generation and barcoding, cleanup, and library preamplification PCR following the Chromium Next GEM Single Cell Multiome ATAC + Gene Expression user guide (10X Genomics; rev. E). The preamplified samples were used to generate the ATAC library and for cDNA amplification to construct the gene expression library.

All libraries generated were subjected to quality control using a 2100 Bioanalyzer (Agilent) before sequencing.

### Multimodal single-cell data alignment and analysis.

The FASTQ files (Babraham Bioinformatics) were quality checked using FastQC and aligned to human genome GRch38 with 10X Genomics’ CellRanger-ARC using the default settings. The counts were loaded into R using Seurat version 4.4.0 and Signac version 1.11.0. Briefly, both the RNA and ATAC counts were loaded into different assays of the same Seurat object. Cells with greater than 1 × 10^5^ or fewer than 1,000 ATAC or RNA counts, a nucleosome signal greater than 2, a transcription start site (TSS) enrichment score less than 1, and percent mitochondrial reads greater than 50 were all removed. ATAC peaks were called using MACS2 version 2.2.7.1 (72), while RNA counts were transformed using SCTransform, regressing out markers of S and G2/M cell cycle phases. PCA on the RNA counts was performed and a value of 20 dimensions was chosen to perform UMAP visualization and clustering. The ATAC peak counts were normalized using term frequency–inverse document frequency and visualized using latent semantic indexing (LSI) and 20 dimensions. WNN clustering was used to integrate both RNA-Seq PCA and ATAC-Seq LSI data into a single UMAP dimensional reduction. For the epithelial cell–enriched sample, 2 small outlier clusters representing nonepithelial contaminants (as determined by *EPCAM* expression) were removed before the sample was reclustered. After identifying cell clusters by both the RNA- and ATAC-Seq data, cluster-specific peaks were called again using MACS2 and cluster-enriched peaks were identified using the FindAllMarkers function. The ATAC bam files were split using the cluster IDs and Sinto version 0.10. Using these peaks and the split bigwig files for the ATAC data, a heatmap was constructed using deepTools version 3.5.1 (73). Motif scanning was performed using ChromVar (53). Lastly, Adaptively Thresholded Low-Rank Approximation gene expression imputation was used to eliminate technical zeroes while preserving biological ones in the epithelial cell–enriched sample (48).

### Bulk RNA-Seq sample and library preparation.

Cells were seeded into a treated 6-well plate at 2 × 10^5^ cells/well density; 48 hours later, RNA was extracted in the same manner as for RT-qPCR. The RNA-Seq library was constructed using a NEBNext rRNA Depletion Kit (NEB; catalog E6310) and a NEBNext Ultra II RNA Library Kit for Illumina (NEB; catalog E7775) according to the manufacturer’s recommendations before being pooled and sequenced using a NextSeq 550 sequencing system from Illumina.

### scRNA-Seq sample library preparation and analysis.

OVCAR4 cells were seeded into a 6-well plate and treated with either an IC50 concentration of cisplatin (2 μM) or the equivalent volume of DMSO (vehicle) for 24 hours. RNA was then collected using TRIzol (Thermo Fisher Scientific; catalog 15596026) according to the manufacturer’s instructions, and the single-cell library was constructed using a 10X Chromium 3′ Single Cell Gene Expression Kit according to the manufacturer’s instructions. Libraries were indexed, pooled, and sequenced on the same lane of a Hi-Seq Next-Gen sequencer. FASTQ files were demultiplexed using CellRanger, and the quality was checked using FastQC. The FASTQ files were aligned to GRch38 using CellRanger with default settings. Analysis was performed as described above.

### In vivo experimental procedure.

Thirty athymic nude female mice (strain 490; Charles River), housed at most 5 to a cage, were subcutaneously injected with 2 × 10^6^ OVCAR4-VPR cells in complete medium mixed with an equal volume of Matrigel Matrix (Corning; catalog 354277). Each mouse received 2 of these injections, 1 on either flank. The right flank received cells expressing the NT sgRNA, and the left flank received cells expressing the SOX9-sg1 sgRNA. The mice were allowed to recover for 1 week before measurements began. On day 7, we began measuring tumor diameters on a weekly basis using digital calipers. Tumor volume was calculated by assuming V = π × ((d/2)^3^). On day 19, the mice received their first treatment dose. Randomly selected cages received either 10 mg/kg carboplatin (*n* = 15) or the equivalent volume of PBS (*n* = 15) i.p. We continued these treatments (on days 26, 33, and 40) while continuing to collect measurements. On day 55, the mice were euthanized, and the tumors were removed, flash frozen, and stored in liquid nitrogen.

### Protein lysate isolation from mouse tumor tissue.

Frozen tumors were removed from the liquid nitrogen storage, wrapped in aluminum foil, and carefully crushed to a powder using a heavy weight. The powder was transferred to a fresh tube and suspended in 2 mL of 1× RIPA buffer (Boston BioProducts; catalog BP-115) with protease inhibitor cocktail tablet (Thermo Fisher Scientific; catalog A32963; 1 tablet/50 mL buffer). The tumor was then blended using a handheld homogenizer until any large pieces were completely broken down. Next, the homogenized tumor suspensions were incubated at 4°C, agitating on a rocker, for 2 hours. Finally, the tumor samples were centrifuged at 16,000*g* for 20 minutes at 4°C, and the supernatants were collected and stored at –80°C for downstream applications.

### Statistics.

All experiments were performed in at least biological triplicates. When comparing 2 groups, 2-tailed Student’s *t* tests were used with 2-stage step-up Benjamini, Krieger, and Yekutieli correction for multiple comparisons. When multiple comparisons were needed against a single control, 1-way ANOVA was used with 2-stage step-up Benjamini, Krieger, and Yekutieli correction for multiple comparisons. All RNA-Seq data were compared using Wilcoxon’s signed-rank tests and reported using FDR to account for multiple comparisons. Significance thresholds used were *P* < 0.05 and FDR < 0.05.

### Study approval.

All animal experiments and procedures complied with ethics regulations of the IACUC of Northwestern University under approved protocol IS00023155.

### Data availability.

The longitudinal single-cell dataset is available through the GEO under accession number GSE165897. Multimodal single-cell data generated for this study are also available through the GEO under accession numbers GSE297350 and GSE296599. All code used to generate figures is available on the MA lab GitHub page at github.com/adlilab/SOX9_Duval_et_al (d940bca247eca099d77a3377d4c5affa64ca31a1). Supporting data are provided in the Supporting Data Values file.

## Author contributions

MA and AJD conceptualized the study and wrote the manuscript. FSP and MR performed single-cell multiomics preparation. FSP also contributed to in vivo experiments. YZ contributed to spheroid assays. MK contributed to single-cell analysis. EJT, DM, SO, AEW, and AEG contributed to tissue acquisition and clinical discussions of the findings. DM provided Kuramochi and COV362 cell lines. ON and ZL contributed to revisions.

## Supplementary Material

Supplemental data

Unedited blot and gel images

Supporting data values

## Version 1 10/01/2025

Electronic publication
