# Supplementary material for: SOX9 drives a stem-like transcriptional state and platinum resistance in high-grade serous ovarian cancer
Source: J Clin Invest. 2025 Oct 1;135(19):e186467. doi: 10.1172/JCI186467 (PMC12483608; doi:10.1172/JCI186467)
Supplement: Supplemental data [file jci-135-186467-s228.pdf]

Supplementary Figures:

Supplement Figure 1

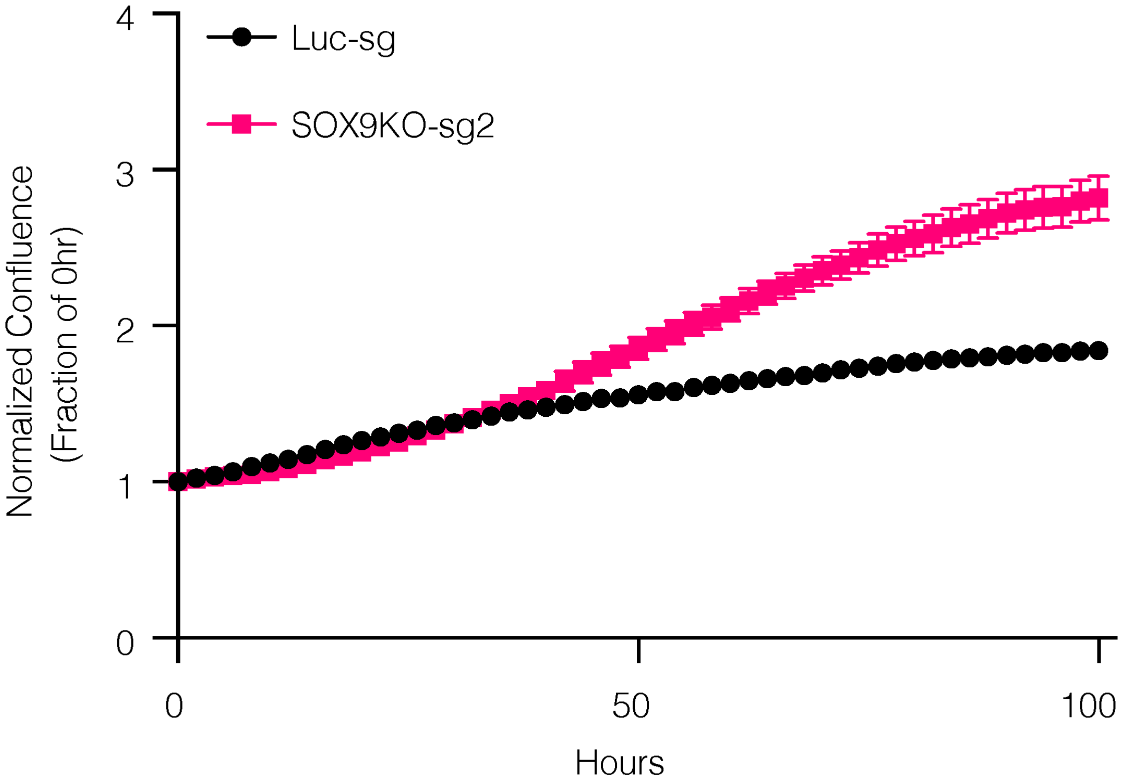

Supplement Figure 1: IncuCyte live-cell imaging measurements of % confluence (normalized fraction of 0h) of OVCAR4 cells with Cas9 and either a Luciferase (NT) sgRNA or a SOX9-KO sgRNA. Error bars shown as mean ± SEM, n=3.

Supplement Figure 2

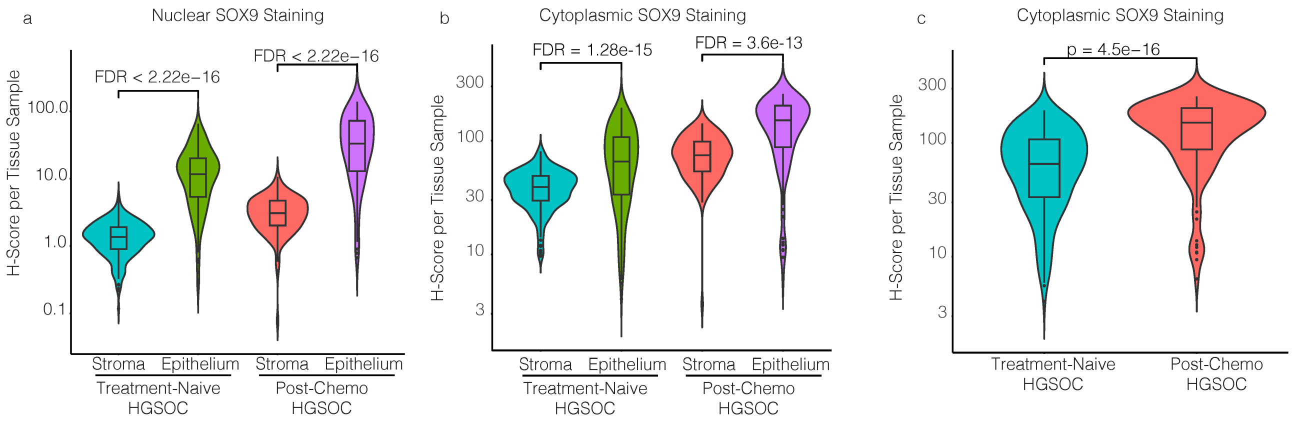

Supplement Figure 2: a) Violin plots depicting DAB-nuclear staining h-scores for epithelial and stromal cells in HGSOC separated by treatment status. b) Violin plot depicting DAB-cytoplasmic staining h-scores for both epithelial cells and stroma in HGSOC separated by treatment status. c) Violin plot depicting DAB-cytoplasmic staining h-scores for epithelial cells only in HGSOC separated by treatment status. P-values and FDR were calculated using a Wilcox signed-rank test. Box and whiskers plots shown depict median, interquartile range, and data range.

Supplement Figure 3

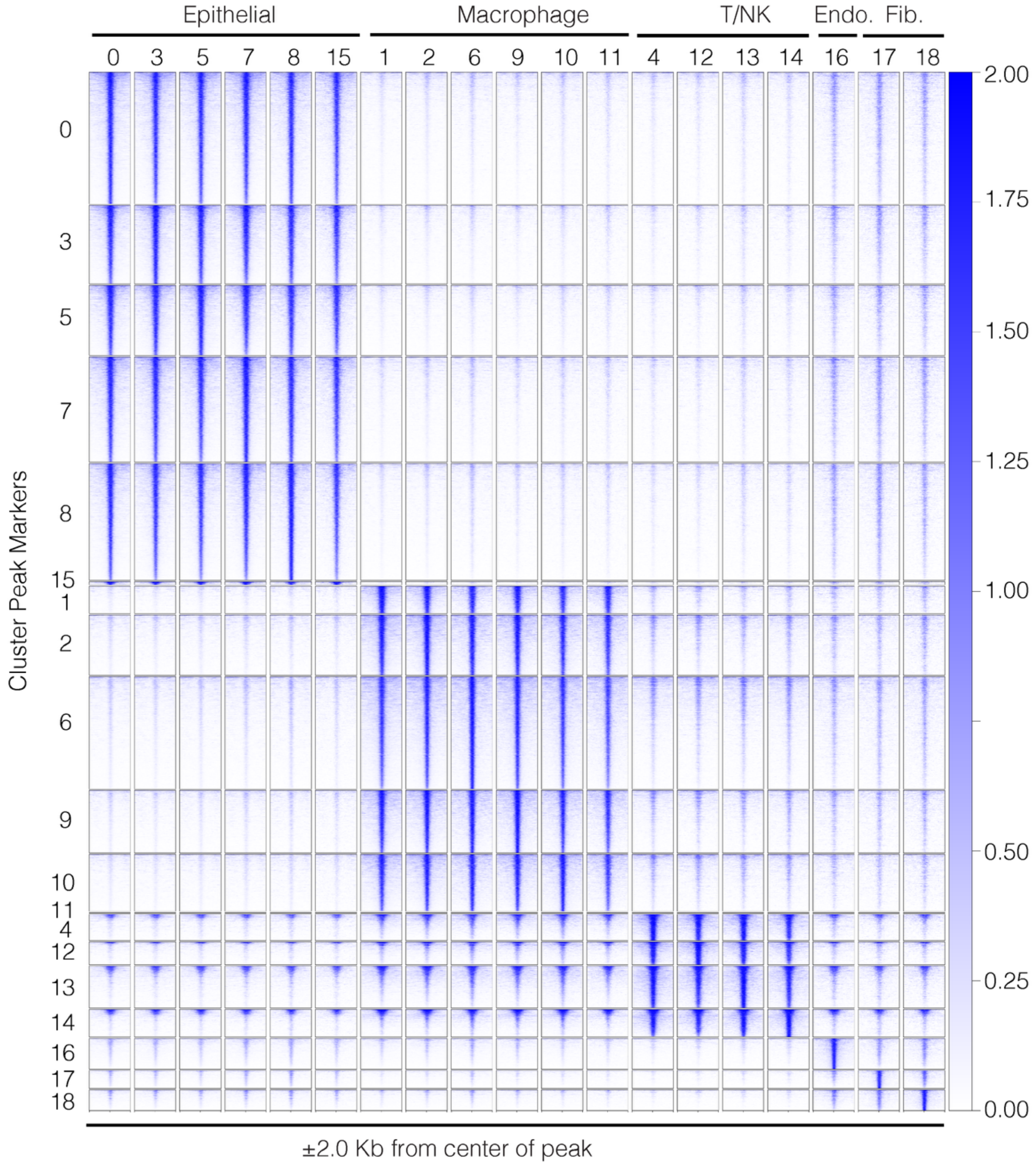

Supplement Figure 3: Heatmap depicting cluster-specific ATAC-peaks (Wilcoxon $p<0.05$) for every cell in the whole tissue HGSOC multimodal snRNA/snATAC-seq dataset.

Supplement Figure 4

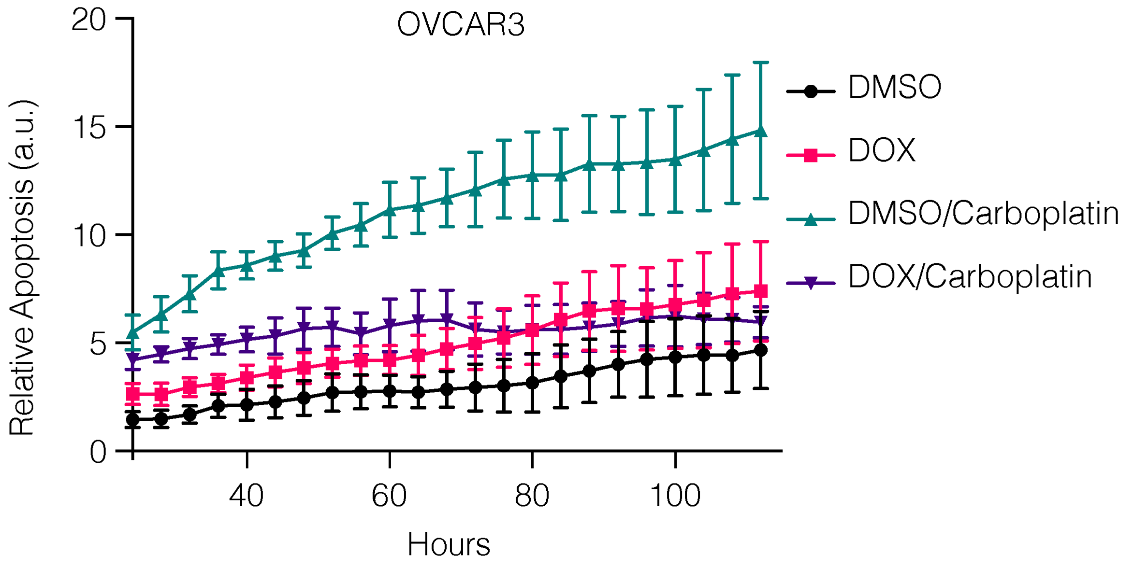

Supplement Figure 4: Relative apoptosis of OVCAR3 cells treated with 0.1 μg/mL DOX or the equivalent volume of DMSO with and without 10 μM carboplatin treatment. Measured using IncuCyte live-cell imager tracking green Caspase 3/7 dye and normalizing to % confluence. Error bars shown as mean ± SEM, n=3.

Supplement Figure 5

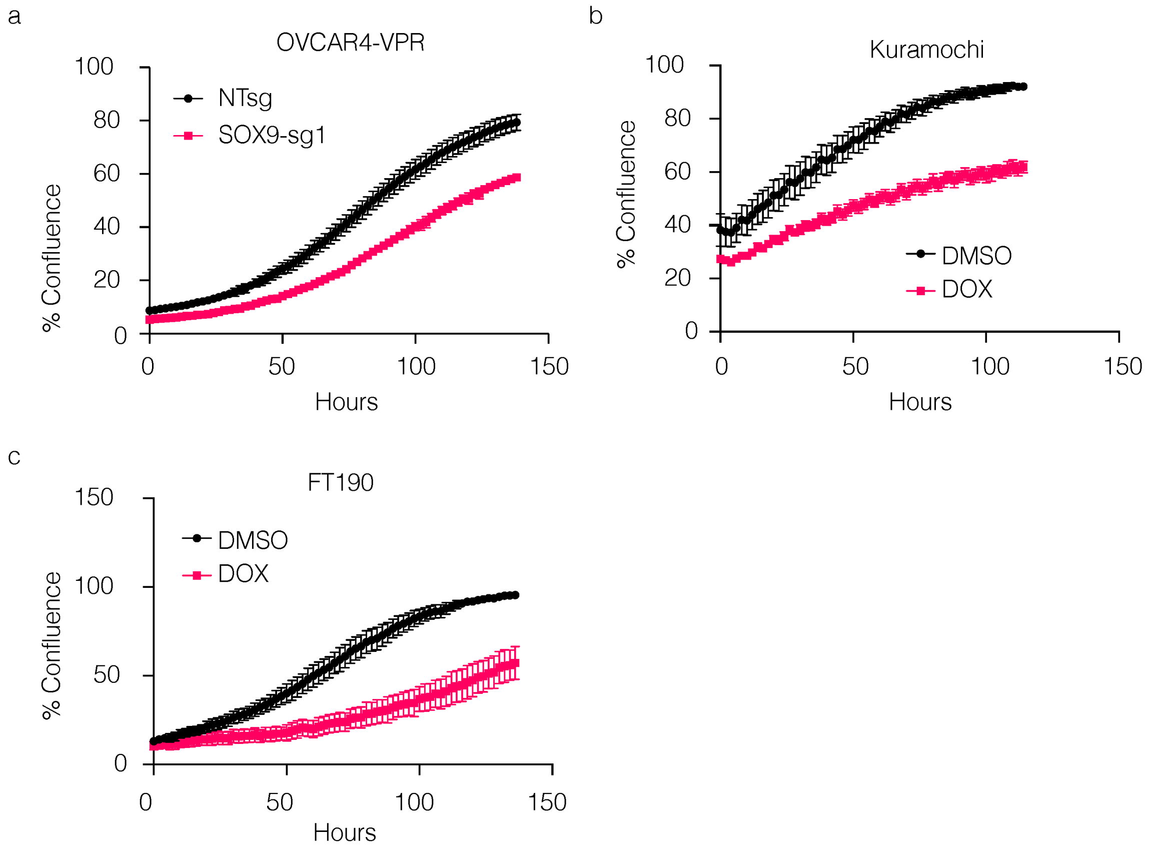

Supplement Figure 5: a-c) % Confluence of cell lines over time as measured by IncuCyte live-cell imager taking images every 2 hours. OVCAR4 (a) cells are shown with epigenetic upregulation of SOX9 (dCas9-VPR + SOX9-sg1) or with a NT-sgRNA. Kuramochi (b) and FT190 (c) cells are shown with the DOX-inducible SOX9 expression system and were treated with either 0.1 µg/mL Doxycycline or the equivalent volume of DMSO. Error bars shown as mean ± SEM, n=3.

Supplement Figure 6

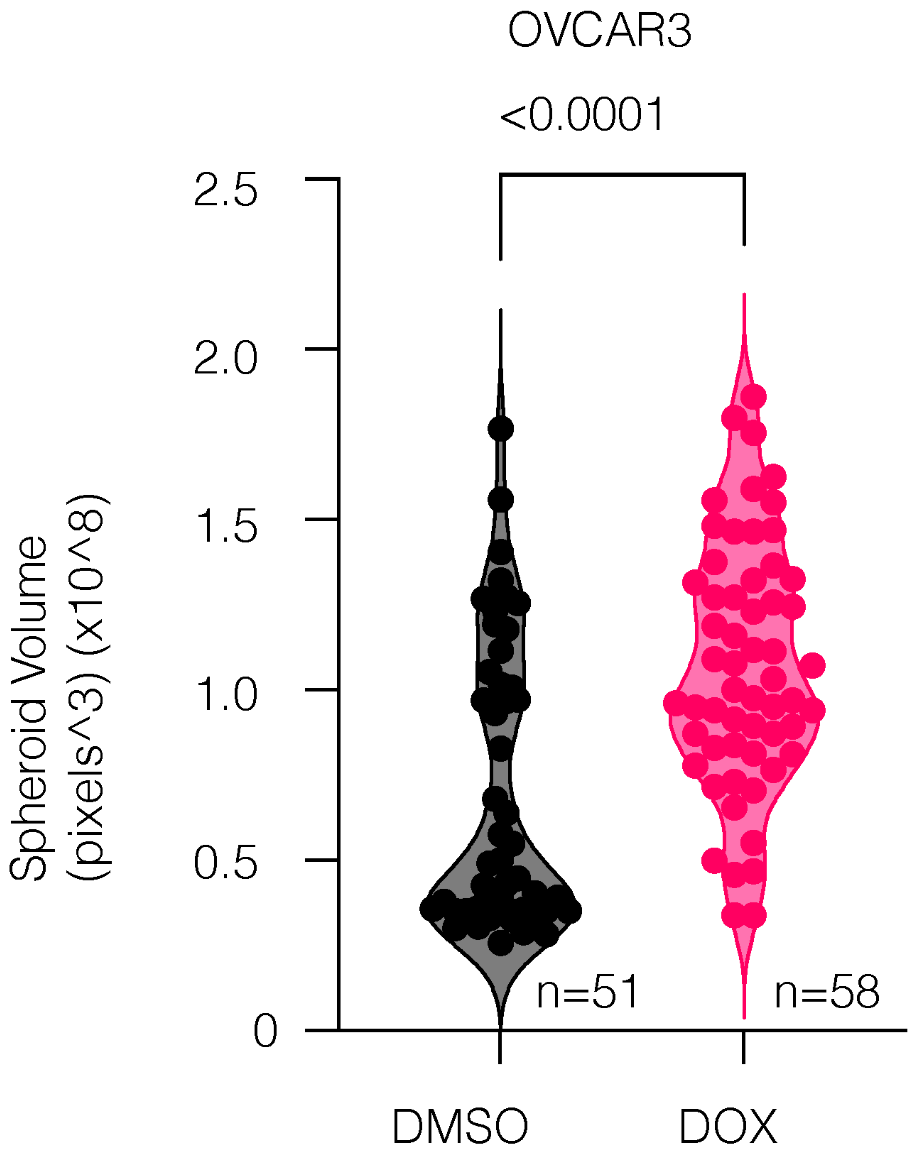

Supplement Figure 6: Hanging droplet spheroids of OVCAR3-FUW-tetO-SOX9 cells treated with 0.1 µg/mL Doxycycline or the equivalent volume of DMSO. Sizes are shown in pixels^3 x 10^8 and the p-value was calculated using an unpaired, two-tailed Student's T-test.

Supplement Figure 7

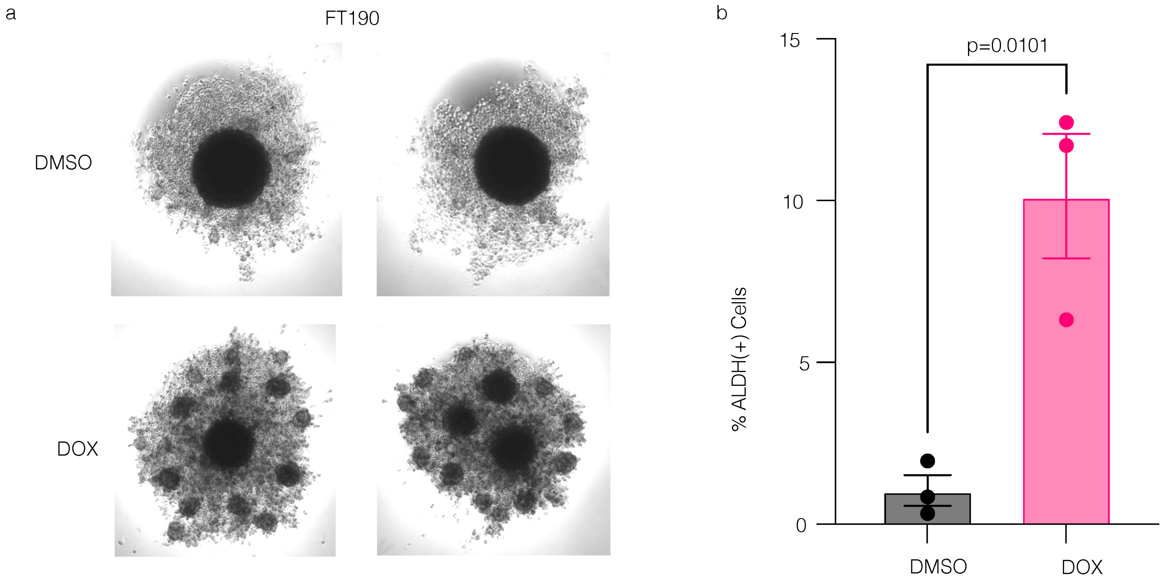

Supplement Figure 7: a) Example hanging droplet spheroids of FT190 immortalized FTE cells containing the DOX-inducible SOX9 expression cassette and treated with either 0.1 µg/mL Doxycycline or the equivalent volume of DMSO. Spheroid images were captured after 7 days growth. b) % ALDH (+) cells as measured using an Aldefluor kit and Flow Cytometry. P-value calculated using two-tailed Student's T-test. Error bars shown as mean ± SEM, n=3.

Supplement Figure 8

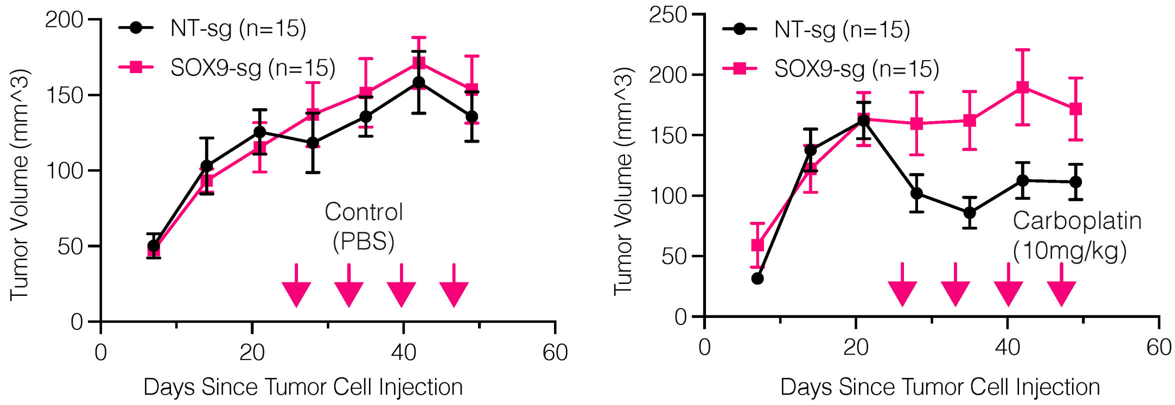

Supplement Figure 8: Absolute tumor volumes from in vivo experiment over time as measured using digital calipers. Error bars shown as mean ± SEM, n=3.

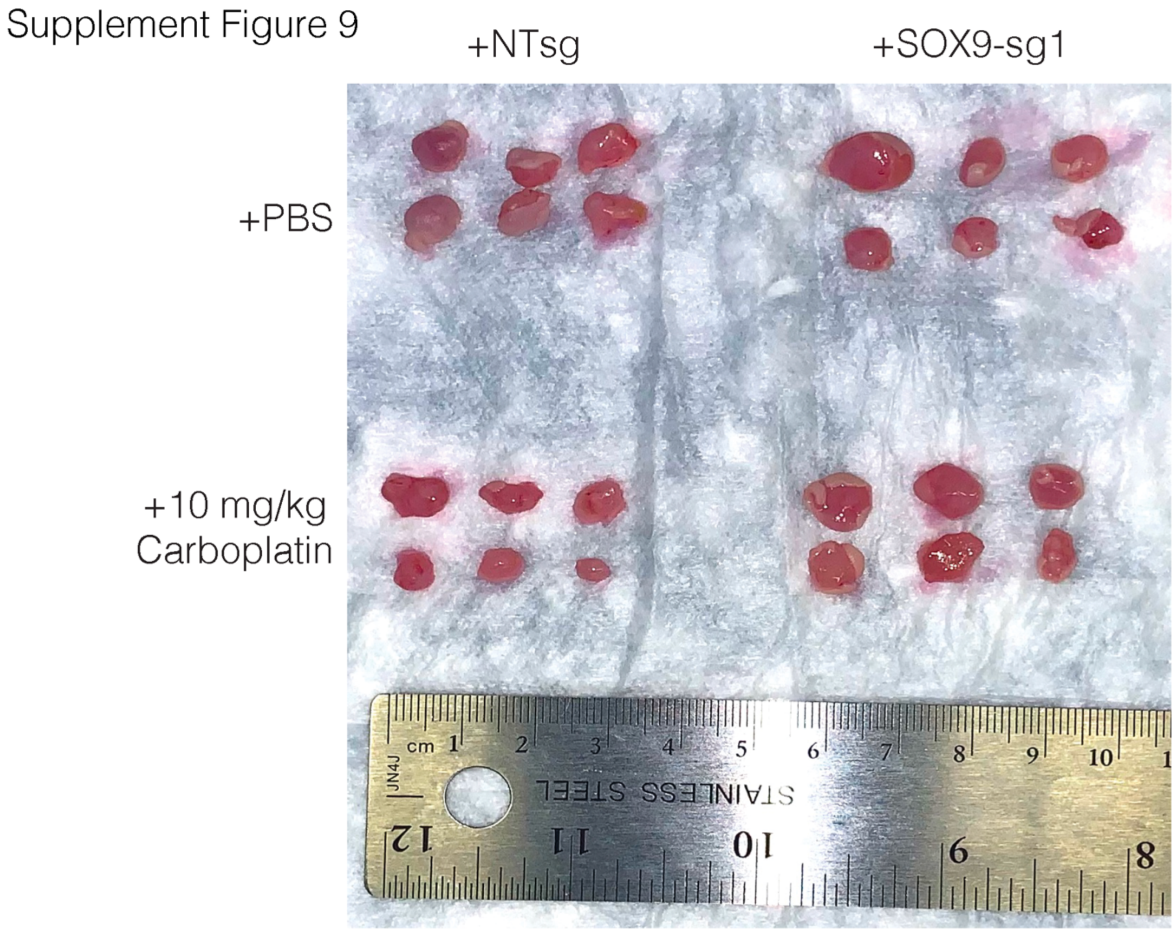

Supplement Figure 9: OVCAR4-VPR tumors expressing either a NT-sg or SOX9-sg1 sgRNA removed from mice that had received 4 doses of either 10 mg/kg carboplatin or the equivalent volume of PBS. Tumors shown are matched from 12 mice.
