# Supplementary material for: SOX9 drives a stem-like transcriptional state and platinum resistance in high-grade serous ovarian cancer
Source: J Clin Invest. 2025 Oct 1;135(19):e186467. doi: 10.1172/JCI186467 (PMC12483608; doi:10.1172/JCI186467)

Figure 1c Full Blots: From left to right: OVCAR4 Parental, OVCAR4 Parental + 20µM Carboplatin (72hrs), Kuramochi Parental, Kuramochi Parental + 20 µM Carboplatin (72hrs), COV362 Parental, COV362 Parental + 20 µM Carboplatin (72hrs). Top blot is SOX9, bottom blot is B-actin.

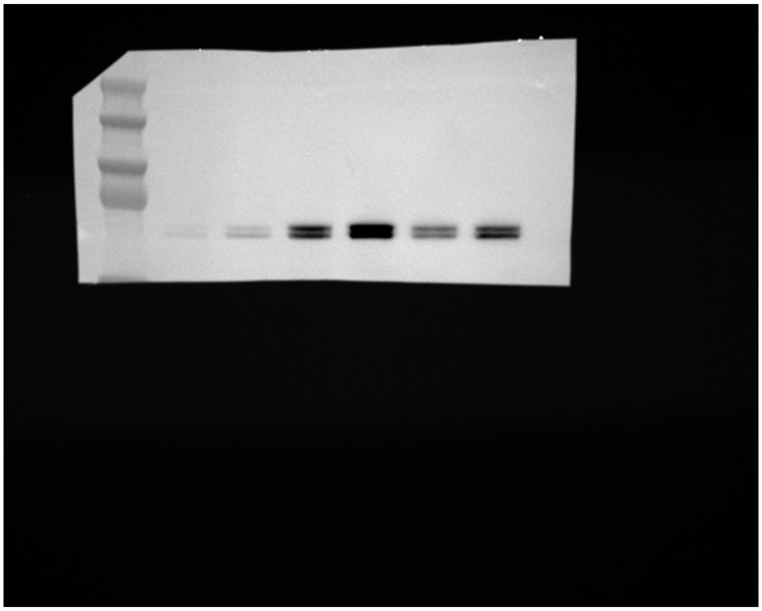

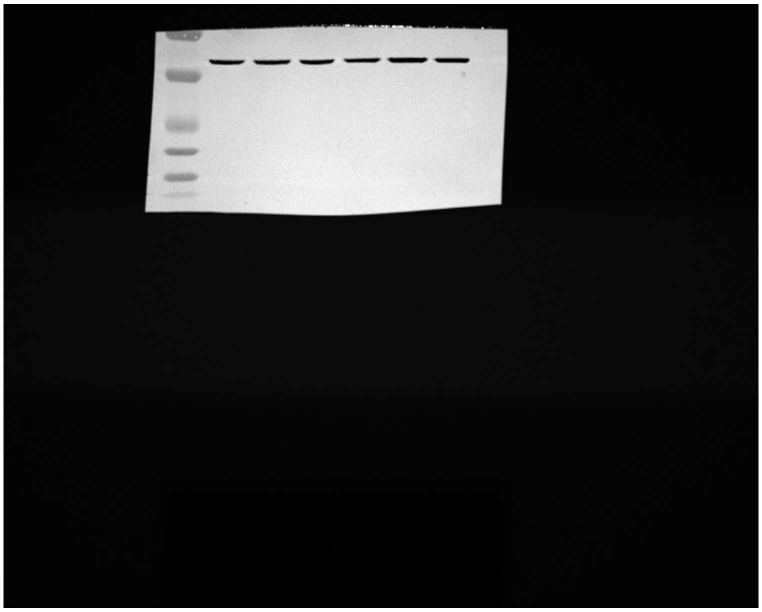

Figure 1e Full Blots: From left to right: OVCAR4 Parental, OVCAR4 + Cas9 + Luc sg, OVCAR4 + Cas9 + SOX9KOsg, OVCAR4 + Cas9 + SOX9KOsg #2 (not used). Top blot is SOX9, bottom blot is B-actin.

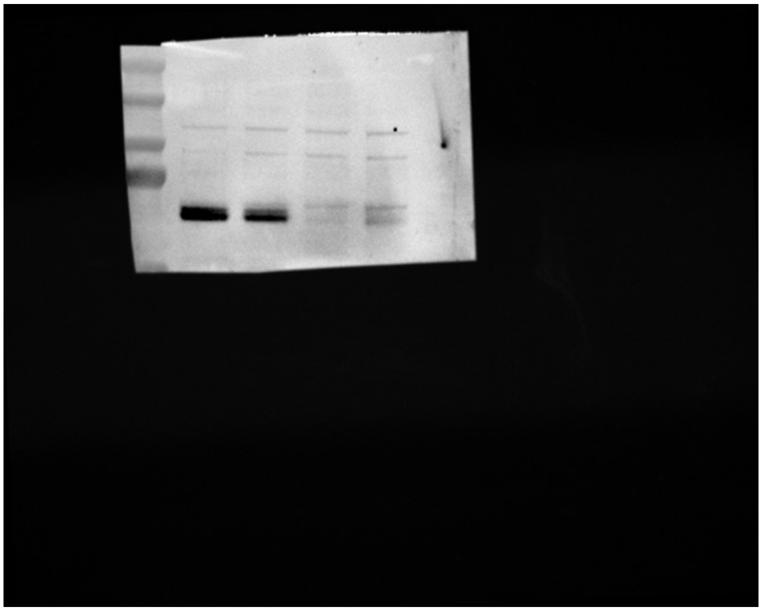

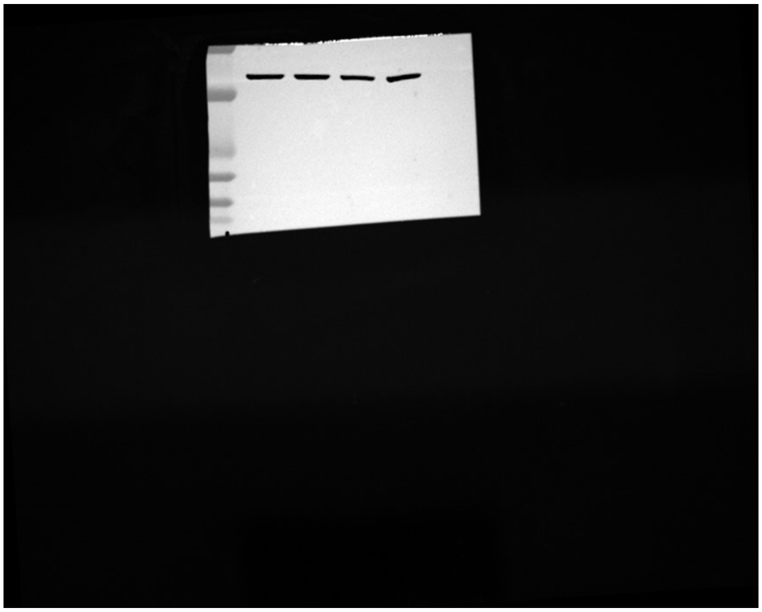

Figure 5a Full Blots: From left to right: OVCAR4 Parental (not used), OVCAR4 Platinum Resistant (not used), OVCAR4-SOX9OE + DMSO, OVCAR4-SOX9OE + 4 ng/mL DOX, OVCAR4-SOX9OE + 8 ng/mL DOX, OVCAR4-SOX9OE + 16 ng/mL DOX, OVCAR4-SOX9OE + 32 ng/mL DOX, OVCAR4-SOX9OE + 64 ng/mL DOX. Top blot is SOX9, bottom blot is B-actin.

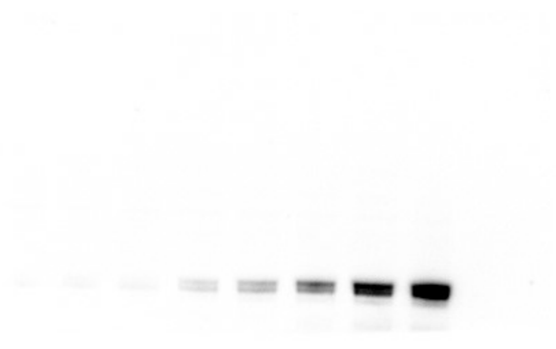

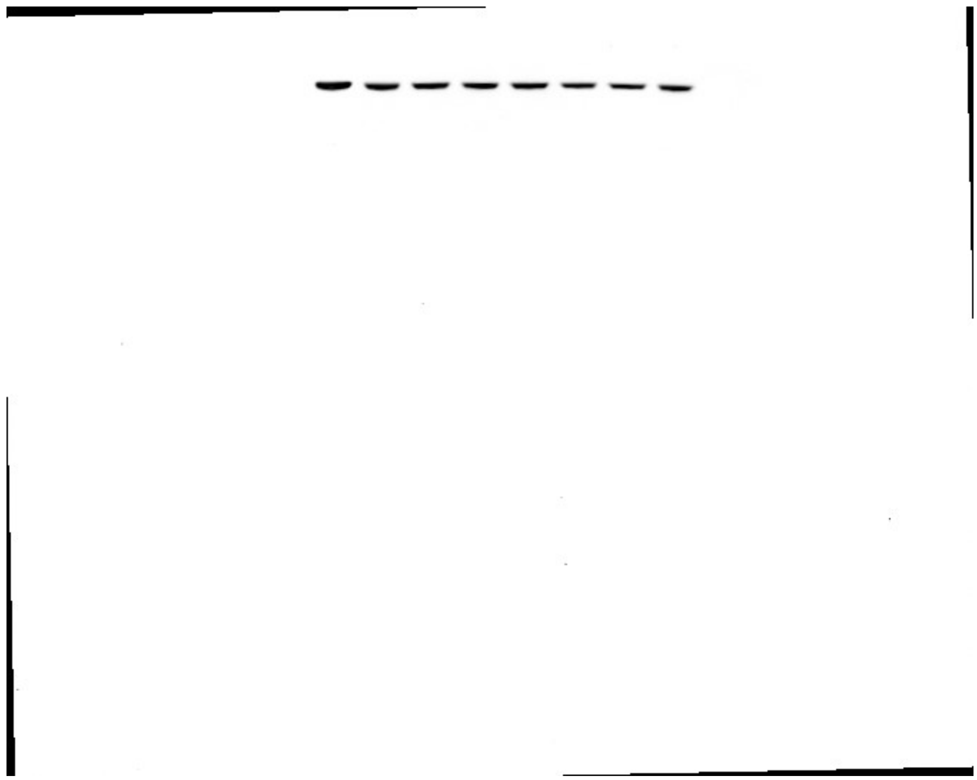

Figure 6f Full Blots: From left to right, Mouse 1 (OVCAR4-VPR + NTsg + PBS, OVCAR4-VPR + SOX9sg + PBS), Mouse 2 (OVCAR4-VPR + NTsg + PBS, OVCAR4-VPR + SOX9sg + PBS), Mouse 3 (OVCAR4-VPR + NTsg + 20 mg/kg Carboplatin, OVCAR4-VPR + SOX9sg + 20 mg/kg Carboplatin), Mouse 4 (OVCAR4-VPR + NTsg + 20 mg/kg Carboplatin, OVCAR4-VPR + SOX9sg + 20 mg/kg Carboplatin). Top blot is SOX9, bottom blot is B-actin.

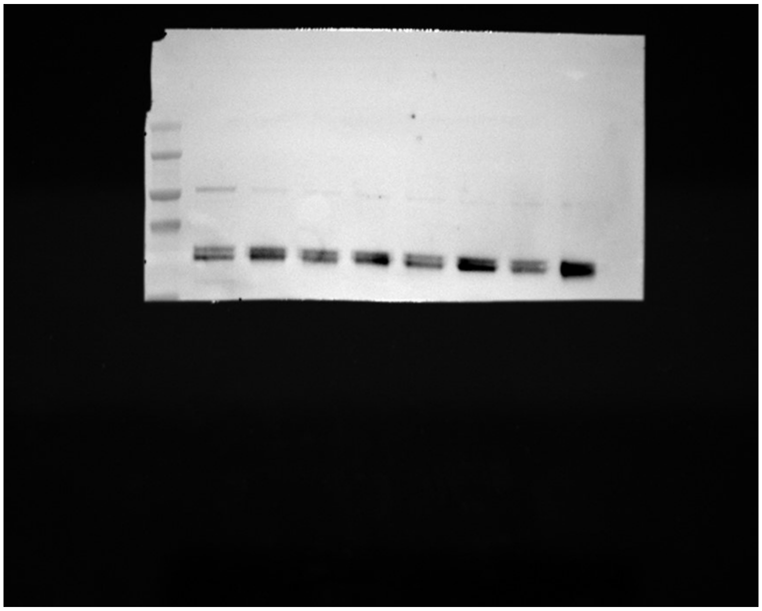

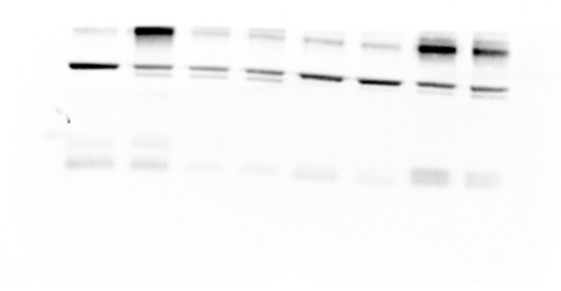

Supplement: Unedited blot and gel images [file jci-135-186467-s229.pdf]
